# Distinct Spectral Profiles of Awake Resting EEG in Disorders of Consciousness: The Role of Frequency and Topography of Oscillations

**DOI:** 10.1007/s10548-023-01024-0

**Published:** 2023-12-29

**Authors:** Dominika Drążyk, Karol Przewrocki, Urszula Górska-Klimowska, Marek Binder

**Affiliations:** 1 Institute of Neurosciences, Université Catholique de Louvain, Brussels, Belgium; 2 Donders Institute for Brain, Cognition and Behavior, Radboud University Nijmegen, Nijmegen, Netherlands; 3 Department of Psychiatry, University of Wisconsin-Madison, Madison, Wisconsin USA; 4 Institute of Psychology, Jagiellonian University, Ul. Ingardena 6, 30-060 Krakow, Poland

**Keywords:** Disorders of consciousness, EEG, Spectral analysis, Resting-state EEG, Coma recovery scale-revised, FOOOF

## Abstract

The prolonged disorders of consciousness (PDOC) pose a challenge for an accurate clinical diagnosis, mainly due to patients’ scarce or ambiguous behavioral responsiveness. Measurement of brain activity can support better diagnosis, independent of motor restrictions. Methods based on spectral analysis of resting-state EEG appear as a promising path, revealing specific changes within the internal brain dynamics in PDOC patients. In this study we used a robust method of resting-state EEG power spectrum parameter extraction to identify distinct spectral properties for different types of PDOC. Sixty patients and 37 healthy volunteers participated in this study. Patient group consisted of 22 unresponsive wakefulness patients, 25 minimally conscious patients and 13 patients emerging from the minimally conscious state. Ten minutes of resting EEG was acquired during wakefulness and transformed into individual power spectra. For each patient, using the spectral decomposition algorithm, we extracted maximum peak frequency within 1–14 Hz range in the centro-parietal region, and the antero-posterior (AP) gradient of the maximal frequency peak. All patients were behaviorally diagnosed using coma recovery scale-revised (CRS-R). The maximal peak frequency in the 1–14 Hz range successfully predicted both neurobehavioral capacity of patients as indicated by CRS-R total score and PDOC diagnosis. Additionally, in patients in whom only one peak within the 1–14 Hz range was observed, the AP gradient significantly contributed to the accuracy of prediction. We have identified three distinct spectral profiles of patients, likely representing separate neurophysiological modes of thalamocortical functioning. Etiology did not have significant influence on the obtained results.

**Supplementary Information:**

The online version contains supplementary material available at 10.1007/s10548-023-01024-0.

## Introduction

The prolonged disorders of consciousness (PDOC) are characterized by a pathological dissociation between wakefulness and conscious awareness. The first is recognized based on eye opening and basic reflexive responses to stimuli. Those behaviors are regulated by subcortical regions including reticular activating system in the brainstem (Demertzi et al. 2009). On the other hand, conscious awareness is characterized primarily by the ability to adequately respond to environmental stimuli and other people, by behaviors such as visual pursuit, following commands or intentional communication. It is believed that the thalamocortical system supports the mechanisms necessary for performing these behaviors (Laureys 2005; Schiff 2010; Gammel et al. 2023). Acute conditions, most frequently following intracerebral hemorrhage, traumatic brain injury, and cardiovascular crisis, may result in a state of coma (Posner et al. 2019) that may further evolve into other forms of PDOC. These include *Unresponsive Wakefulness Syndrome* (UWS, Laureys et al. 2010), which is characterized by the lack of self-awareness or the awareness of environment (Monti et al. 2010), with preserved irregular sleep cycles as well as intact respiration and thermoregulatory capacity (Gosseries et al. 2011). The recovery from UWS may result in a *Minimally Conscious State* (MCS), a state in which the patient is able to follow simple commands, visually pursuit objects and people or respond verbally (Schnakers et al. 2016). However those behavioral manifestations of consciousness may be intermittent in MCS. Reappearance of functional communication and/or ability to use objects adequately is a sign of the *Emergence from Minimally Conscious State* (EMCS, Giacino et al. 2002). The correct differentiation between the above mentioned states remains a great challenge for medical personnel as well as caregivers. The repeatedly observed high rate of misdiagnosis of UWS (Childs et al. 1993; Schnakers et al 2009; Forgacs et al. 2014) only emphasizes the need for improved diagnostic protocols.

Standardized clinical tools, of which the gold standard is *Coma Recovery Scale-Revised* (CRS-R; Giacino et al. 2004; Seel et al. 2010), may reduce the likelihood of diagnostic errors (Schnakers et al. 2009; van Erp et al., 2014). Unfortunately however, their reliability is limited as they are based on the overt motor responses to sensory stimulation and verbal communication. Using them as the only diagnostic tool may result in the understatement of patients neurocognitive capacity, especially in cases of *Cognitive-Motor Dissociation* (CMD, Schiff, 2014; Stender et al. 2014; Schnakers 2020) or *Locked-In Syndrome* (LIS, Gosseries et al. 2016), where motor unresponsiveness prevents patients from overtly following commands.

The non-invasive, relatively low-cost and widely available EEG technique is a promising alternative that may contribute to a better diagnosis. A resting-state EEG signal can be regarded as a rich source of information about the functional integrity of the thalamocortical system (Forgacs et al. 2014). Frequency domain EEG analysis, representing contributions of individual frequencies in the signal, has already been proved very useful in PDOC diagnosis (Bai et al. 2017).

It has been previously demonstrated that the ratio of high (8–30 Hz) to low (2–8 Hz) frequencies correlates with the CRS-R total score (Lechinger et al. 2013). Other studies pointed out that the power of delta range (1–4 Hz) is related to unfavourable outcome in PDOC patients (Lehembre et al. 2012; Piarulli et al. 2016). A good discriminative potential regarding the PDOC diagnosis was also assigned to the *slowing* of oscillatory power within the range of 4–13 Hz in the parietal region (Schiff et al. 2014). Unfortunately, the mechanisms of this phenomenon are not well understood. In most cases EEG data is analyzed by averaging the amplitude within separate frequency bands. Therefore, it is not clear whether the observed effect is related to the shift of a single frequency peak related to increasing level of neurocognitive capacity or, alternatively, it is a result of independent low-frequency oscillatory activity in UWS and middle-frequency alpha-like activity in MCS and EMCS.

Previous studies also investigated differences in spatial dynamics of the patient's spectral profile. For example, the antero-posterior (AP) gradient was used, representing the gradual increase in frequency with the parallel decrease in amplitude observed on the antero-posterior axis of a scalp, including dominant frontal beta activity (Forgacs et al. 2017; Hirsch et al. 2013, 2021; Schiff et al. 2014; Estraneo et al. 2016). This feature illustrates characteristic dynamic differences when it comes to the distribution of alpha rhythm in wakefulness and sleep stages (DeGennaro et al. 2001) and was shown as a promising feature of preserved corticothalamic integrity in PDOC patients (Forgacs et al. 2017).

In this study we investigated the relationship between the frequency peaks of the highest amplitude within the 1–14 Hz range of the frequency spectra and the outcome of the CRS-R behavioral scale in the group of PDOC patients with various etiologies. Moreover, we examined the spatial dynamics of this oscillation, and evaluated the usefulness of AP gradient, previously advocated as a marker of the functional recovery in the PDOC group (Forgacs et al. 2014; Forgacs et al. 2016; Schiff et al. 2014; Estraneo et al. 2016). While the existence and features of AP gradient were previously assessed by the expert qualitative evaluation, this research aims to investigate the possibility of its quantitative operationalization, with a use of FOOOF algorithm (Fitting Oscillations and one over F, Donoghue et al. 2020), a robust and easy-to-use spectral decomposition method. We found that the increase in the dominant peak predicts the increase of the CRS-R total score, while the outcome from the gradient measure depends on the integrity of the dominant peak in the EEG spectrum.

## Materials and Methods

### Subjects

The initial group of healthy controls (HC) included 45 subjects, each with the single EEG measurement. From the initial group 8 subjects were excluded due to the large muscle artifacts and/or an extensive noise in the EEG signal. Thus, the final dataset used in the analysis included 37 measurements obtained from HC (*N* = 37 including 22 females, aged 23.94 ± 3.15).

The initial group of PDOC patients comprised 134 measurements, obtained from 78 patients. Due to the large muscle artifacts and/or an extensive noise present in the EEG centro-parietal electrodes we excluded 49 measurements (see Table 1 for details). This amount, although high (~ 37%), presents no deviation from the common exclusion criteria in the area of research on patients with severe brain injuries (Cao et al. 2019; Pistoia et al. 2014; Kremneva et al. 2019; Forgacs et al. 2017). The high rejection rate primarily stems from the absence of collaboration and the neurological status of the patients. This status results in abnormal posturing associated with an excessive muscle tone, frequently encompassing the muscles of the head and neck. Other factors include frequent uncontrolled spasms, sweating, nystagmus, and atypical blinking patterns. Finally, the analysis included a set of 86 measurements from 60 PDOC patients (*N* = 53, including 20 females, aged 36.88 ± 13.53, see Table 1 for details).

**Table 1:** Demographic features of groups of patients within a CRS-based diagnosis

CRS-based diagnosis					
Groups	N measurements	N	Females	Age	
				M	SD
UWS	30	22	5	36.33	12.43
MCS	36	25	12	39.31	15.47
EMCS	20	13	7	32.56	10.01

*UWS* unresponsive wakefulness syndrome, *MCS* minimally conscious state, *EMCS* emergence from MCS

Table A1 in Supplementary Material provides detailed information about the PDOC patients, including the sex, age, etiology, CRS-R results with the subscales and diagnosis, time since injury in months, dates of EEG and CRS measurements, list of administered medications, and finally the exclusion criteria, when applicable.

An informed consent was obtained from each healthy participant and from the legal surrogates of all patients. The study was approved by the ethics review board of the Institute of Psychology, Jagiellonian University and was conducted in accordance with the Declaration of Helsinki (1975, revised 2000).

### EEG Measurement

PDOC resting state EEG data was collected in three specialized in-patient centers for care and treatment in Toruń (Fundacja "Światło"), Częstochowa (COiR "Zdrowie") and Kraków (PCRF "Votum"), Poland. Healthy control group data were collected in the Psychophysiology Laboratory of the Institute of Psychology, Jagiellonian University in Krakow, Poland. The recording was performed using 64-electrode Active Two (BioSemi, Amsterdam, NL), with a 10–20 system headcap and four additional electrodes located above and below the right eye and in the external canthi of both eyes. Two additional reference electrodes were placed on mastoids and recorded in parallel. ‘CMS’ (*common mode sense*) and ‘DRL’ (*driven right leg*) electrodes were placed between ‘POz’ and ‘PO3’ and ‘POz’ and ‘PO4’, respectively.

For each measurement, the recording was carried on for 10 min and sampled at 1024 Hz. Patients were examined in the isolated room, reclining in bed or sitting on the wheelchair in a comfortable position, with their eyes open. Eye opening was consistently monitored for each patient during EEG acquisition. In the event of eye closure, an arousal maintenance protocol (based on CRS-R guidelines) was implemented. If re-establishing eye opening was not feasible, the recording was terminated, and the data were excluded. Healthy control group was examined when sitting on a chair in the sound isolated laboratory room, with their gaze set on the fixation cross in the center of the computer screen, and were instructed not to focus on anything specific during the recording time.

### Preprocessing

The off-line preprocessing of EEG data was performed with Brain Vision Analyzer 2.0 software (Brain Products, Gilching) in the following order. First, noisy channels were interpolated with a spline method. Next, data was filtered in 1–50 Hz range (IIR Filter, Zero phase shift Butterworth filter; order 8), and downsampled to 256 Hz. Then, raw data inspection excluded massive artifacts before the segmentation. In the next step blink and eye movement artifacts were corrected using Infomax Independent Component Analysis (ICA). Data was further re-referenced to the common average. Next, signal from each channel was segmented into non-overlapping 2 s intervals, with the noisy segments excluded from the analysis using semi-automatic mode with the following criteria: amplitude limits − 150 μV to 150 μV; 150 μV maximum allowed difference in intervals over 200 ms; maximal voltage step of 75 μV/ms. Finally, Fast Fourier Transform (FFT, 10% Hanning window, 0.5 Hz resolution) was calculated on the remaining segments and averaged, for each channel separately.

Due to frequent excessive movements, posturing and muscle twitches and structural brain damage, EEG data acquired from PDOC patients may become noisy, yet the signal from parietal and central regions of the scalp is usually the least affected by those artifacts (Forgacs et al. 2020). Thus, the *centro-parietal* Region Of Interest* (centro-parietal ROI)*, constructed by averaging the electrodes ‘Cz’, ‘CPz’, ‘Pz’, ‘CP3’, ‘CP1’, ‘P3’, ‘P1’, ‘CP2’, CP4’, ‘P2’, and ‘P4’, was used to calculate the frequency of the highest peak in the EEG spectrum. Further, individual activity of the ‘POz’, ‘Pz’ and ‘CPz’ electrodes was used to calculate *midline AP gradient*. We did not include the frontal channels (e.g. Fz or FCz) due to the high frequency of artifacts in those channels.

### Data Analysis

The clinical state of the patients was determined using the Polish adaptation of the *Coma Recovery Scale-Revised* (CRS-R; Binder et al. 2018). CRS-R is the most recommended clinical tool for behavioral assessment of neurocognitive functions in PDOC patients (Giacino et al. 2009; Schnakers 2020). It consists of 23 items hierarchically arranged into six subscales that address auditory, visual, motor, oromotor, communication and arousal functions, so that within each subscale the lowest score corresponds to the reflexive responses, and the highest score indicates the presence of a cognitively mediated behavior (Giacino et al. 2004). The experimenters were blind to the results of the CRS-R assessment at the time of recording and data preprocessing.

In the analysis described below, the total score of the CRS-R scale obtained for each patient is represented with the *CRSscore* variable, while the PDOC diagnosis obtained on the basis of individual CRS-R results (UWS, MCS or EMCS) with the *CRSdiagnosis* variable.

The averaged EEG spectra were parameterized using FOOOF algorithm (version 0.1.3) with the *fooof_mat* wrapper (version 0.0.1, Donoghue et al. 2020) within the MATLAB environment (Mathworks Inc., version 2019b). The method allows for a precise isolation of frequency peaks in the EEG signal spectrum, while controlling for the background aperiodic signal component. FOOOF algorithm applies a linear fit to the spectrum in log–log space and subtracts the obtained linear trend from it. Further, a Gaussian fit is iteratively fitted to the largest peak that exceeds the assumed threshold. The largest peak is then removed from the spectrum and the threshold recalculated for the next iteration. The procedure ends when no peak exceeding the relative threshold is found and a model of the aperiodic part is created. Thus, FOOOF algorithm allows not only for the extraction of the aperiodic part of the spectrum, but also the analysis of the parameters of the periodic part: center frequency of Gaussian peaks, their amplitude and bandwidth (Donoghue et al. 2020). The goodness of fit metrics are represented by R^2^ of the model fit and an error estimate. The FOOOF algorithm was implemented using the following settings. Maximal number of peak fit iterations was restricted to 4. Modeling was executed using fixed aperiodic mode. Limits of the Gaussian peak width were set to 2–6 Hz. Minimum peak height was set to 0.111 μV. Relative peak threshold was restricted to 2 relative units of power spectrum. Power spectra were parameterized across frequency range between 1 to 45 Hz.

Based on FOOOF results, for each measurement we obtained a *MaxPeakFreq* parameter, which was the center frequency of the peak with the highest amplitude within the 1–14 Hz search range (extending across delta, theta and alpha EEG frequency bands) in the averaged signal from the centro-parietal ROI. Importantly, in contrast to Forgacs et al. (2017), our analysis was concentrated solely on the 1–14 Hz frequency range. The noise level in the beta range of our data, primarily attributed to muscle activity, precluded us from drawing reliable conclusions. Furthermore, as the literature review in the Introduction indicates, the lower bands provide the most pertinent information regarding the status of PDOC patients.

The *Gradient* parameter, representing the antero-posterior (AP) gradient of the amplitude of the highest peak with the central frequency in range 1–14 Hz, was calculated as follows: for the 1–14 Hz range of modeled power spectrum of each of the midline electrodes ‘POz’, ‘Pz’ and ‘CPz’, we found the amplitude of the highest peak. To make sure we are tracking the same peak, as it has been determined by the *MaxPeakFreq*, every other peak deviating from the *MaxPeakFreq* by at least half of its bandwidth on each side of the spectrum was rejected. For further analysis we used only those measurements in which the specified peak was successfully found in at least 2 of 3 midline electrodes, with at least 0.95 goodness of modeled power spectrum fit. Then we constructed a vector of peaks amplitudes, normalized by dividing each value by the maximal value of the vector. Finally, to obtain *Gradient* parameter we calculated vectors gradient, using *gradient* function (MATLAB ver. R2019b) and averaged it across three previously selected channels.

### Statistical Analysis

To capture the relationship between the resting-state EEG measures and the patient neurobehavioral condition assessed using CRS-R scale, we introduced two separate models: *Model 1* with a total CRS-R score (labeled further as *CRSscore*) and *Model 2* with a CRS-R based diagnosis (labeled further as *CRSdiagnosis*), as respective outcomes. We adopted the total CRS-R score (range 2–23 with the mean of 10.714 ± 5.941), as the index of the overall level of neurocognitive capacity in PDOC patients (Bodart et al. 2018; Rosanova et al. 2012). The CRS-R-based PDOC diagnosis was treated as an ordinal categorical variable. This variable consisted of four ordered categories: from the group with the lowest (UWS), through intermediate (MCS) and the highest capacity (EMCS), to the healthy control group (HC). The HC group was included to illustrate the contrast between the patterns of resting state activity observed in an intact, aware brain and those with injuries affecting the state of consciousness.

*Model 1.* In the *CRSscore* analysis we decided to follow the multilevel models strategy, as it takes into account subject-level variability better than the traditional methods of data aggregation (Moscatelli et al. 2012). Statistical analysis was performed using the R environment (R Core Team, 2021, version 4.0.3) with *lme4* library (version 1.1-27.1; Bates, Mächler, Bolker, Walker, 2015). Linear Mixed Models (LMMs) were fitted using the maximum likelihood method (Laplace Approximation, Bates, Mächler, Bolker, Walker, 2015). The structure of factors used in the model was constructed step up, from the model with *1/Subject* variable as a random intercept (*Model 1n*), to more complex ones with singular (*Model 1a* with *MaxPeakFreq, Model 1b* with *Gradient*) and both fixed factors (*Model 1c*) included. Random intercept structure in every model controlled for the within-subject variance resulting from the repeated measures design. Log-likelihood (*LL*) as an indicator of model selection was compared for all models, each time contrasting the model with the effect in question against the model without the effect in question. The one with the lowest log-likelihood value was considered final (Snijders and Bosker 2012).

*Model 2.* Probability of the given diagnosis (*CRSdiagnosis*) based on the *MaxPeakFreq* and *Gradient* was calculated using cumulative link mixed models (CLMM, Agresti, 2002) for the ordinal outcomes with *probit* distribution. Analysis was performed using the *ordinal* library (version 2020.8-22; Bürkner and Vuorre, 2019). The structure of factors used in the model was constructed step up, from the model with *Subject* variable as a random intercept (*Model 2n*), to more complex models with single (*Model 2a* with *MaxPeakFreq, Model 2b* with *Gradient*) and both fixed factors (*Model 2c*) included. Random effects structure and model selection process was the same as in *CRSscore* analysis.

## Results

### Total CRS-R Score in Relation to the Frequency of the Maximum Peak in the Spectrum and the Antero-Posterior Gradient

**Fig. 1:**
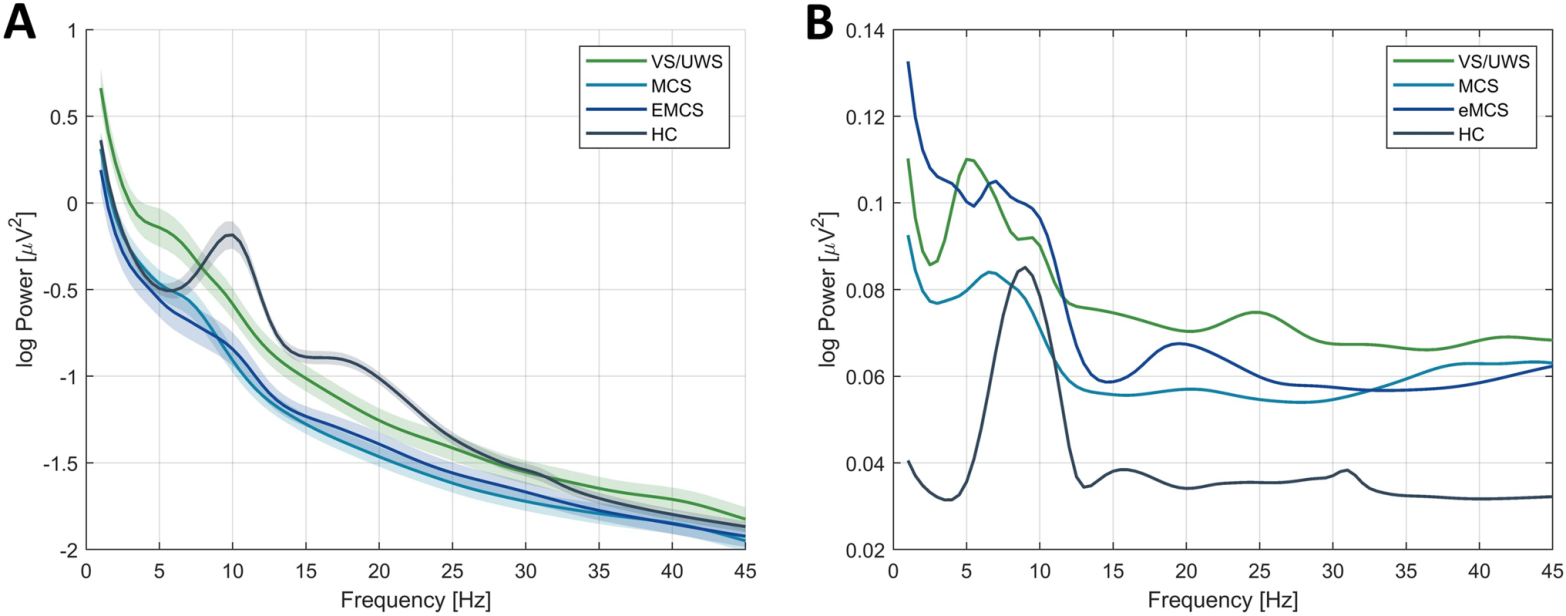
**A** The averaged power spectra (*solid lines*) of the *centro-parietal ROI* modeled with FOOOF parameters. Spectra are presented for all patient PDOC groups (*UWS* unresponsive wakefulness syndrome, *MCS* minimally conscious state, *EMCS* emergence from MCS) and the healthy control group (*HC*). *Coloured ribbon* represents one standard error of the mean (SEM) around the averaged fit. **B** Averaged SEM values from the same *centro-parietal ROI* showing differences in variability of power scores at lower frequencies across the PDOC groups (Color figure online)

**Fig. 2:**
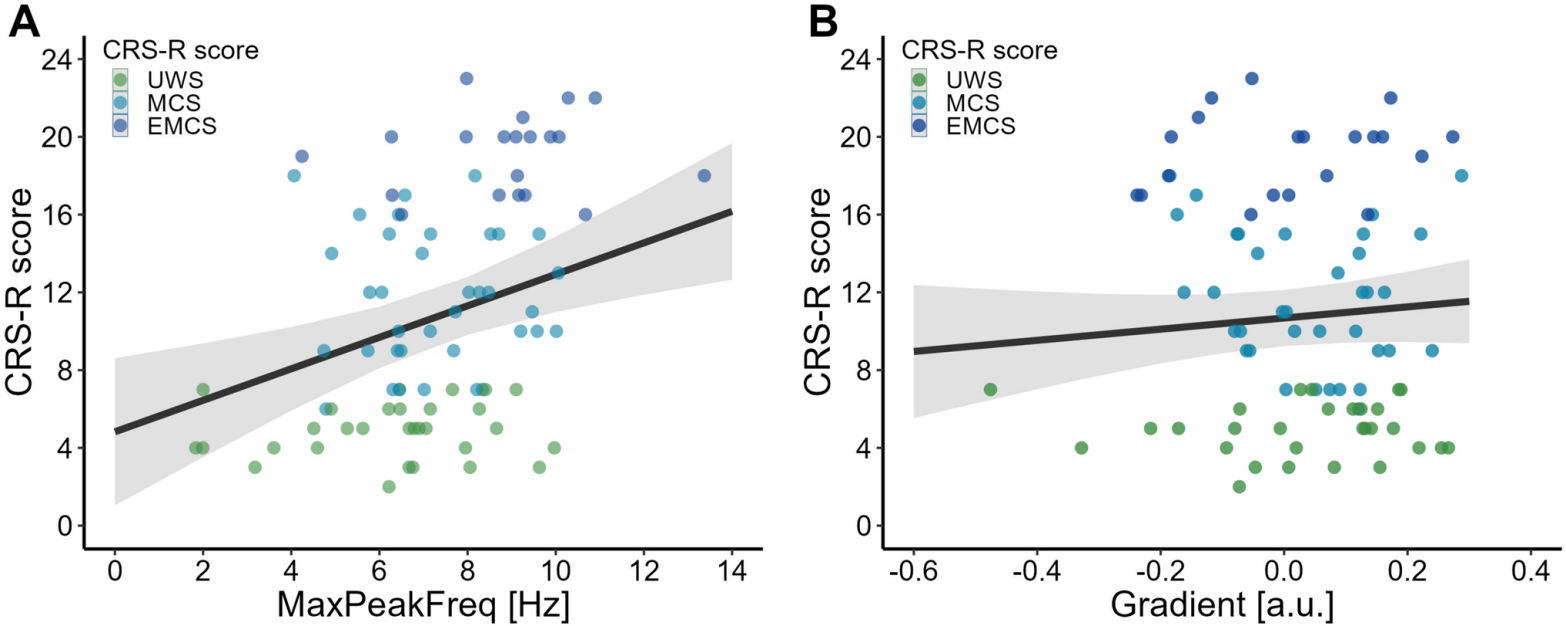
The relationship between CRS-R score value and electroencephalographic parameters. **A** CRS-R score value (*dots*) plotted against the frequency of the maximum peak in range 1–14 Hz (*MaxPeakFreq*). Colors indicate PDOC groups (*UWS* unresponsive wakefulness syndrome, *MCS* minimally conscious state, *EMCS* emergence from MCS). *Black solid line* represents the main effect of *MaxPeakFreq* from Model 1c. *Gray ribbon* represents 95% SE around the fit. **B** CRS-R score value plotted against antero-posterior gradient (*Gradient*). *Black solid line* represents the main effect of *Gradient* from *Model 1c.* *Gray ribbon* represents 95% SE around the fit (Color figure online)

Total CRS-R scores for individual patients are presented in Table A1 (Appendix A, Supplementary Material) and the averaged spectra fitted using FOOOF parameters in Fig. 1A. Standard errors of the mean that depict comparison of power variability across PDOC patient groups are presented in Fig. 1B. *Model 1a* was used to assess the relationship between *CRSscore* and the frequency of the highest peak in the 1–14 Hz range (*MaxPeakFreq*). Model presented better fit compared to the null model (ΔLL = − 5.11, see Table 2). Analysis showed a statistically significant effect of *MaxPeakFreq* (*β* = 0.74 ± 0.22, *CI:* [0.30 1.20], Fig. 2A). On the contrary, *Model 1b* (*LL* = -251.39) with the antero-posterior gradient (*Gradient*) as a variable did not present a significant effect [*CI:* (− 5.19 4.65), Fig. 2B]. Although *Model 1c* including the interaction of *MaxPeakFreq* and *Gradient* was characterized with minimally better fit (*LL* = − 245.69) compared to the *Model 1a*, the improvement was too small to account for the cost of increased complexity of the model. The interaction itself was not significant [*CI:* (− 1.63 2.13)], *Model 1c* was therefore rejected. Results indicate that the increase of *CRSscore* could be related to the increase of the frequency of the highest peak in the 1–14 Hz range, but not to the value of the antero-posterior gradient.

**Table 2:** Summary of the *CRSscore* models fitted with its accuracy diagnostic

Model	Effects	Estimate ± SE	t	p	95% CI	$\sigma^{2}$	$\tau^{00}$	LL
Model 1n	Intercept	10.64 ± 0.80	13.30	** < 0.001**	**[9.12 12.15]**	4.91	30.26	− 251.39
Model 1a	Intercept	5.26 ± 1.78	2.96	**0.004**	**[1.77 8.75]**	4.74	25.08	− 246.28
	MaxPeakFreq	0.74 ± 0.22	3.33	**0.001**	**[0.30 1.17]**			
Model 1b	Intercept	10.64 ± 0.80	13.29	** < 0.001**	**[9.08 12.22]**	4.90	30.30	− 251.39
	Gradient	− 0.22 ± 2.56	− 0.08	0.932	[− 5.14 5.04]			
Model 1c	Intercept	4.81 ± 1.95	2.46	**0.016**	**[0.92 8.85]**	4.82	24.11	− 245.73
	MaxPeakFreq: Gradient	0.25 ± 0.98	0.26	0.797	[− 1.84 2.31]			

Results that are significant (p < 0.05) are indicated in bold font

*LL* Log-Likelihood, $\sigma^\textit{2}$ variance of level-1 residual errors, $\tau^\textit{00}$ variance of level-2 residual errors

### Evaluation of Diagnostic Utility of the AP Gradient and the Frequency of the Maximum Peak

The size of each of four groups included in the analysis, together with its demographic statistics is presented in Table 1.

**Fig. 3:**
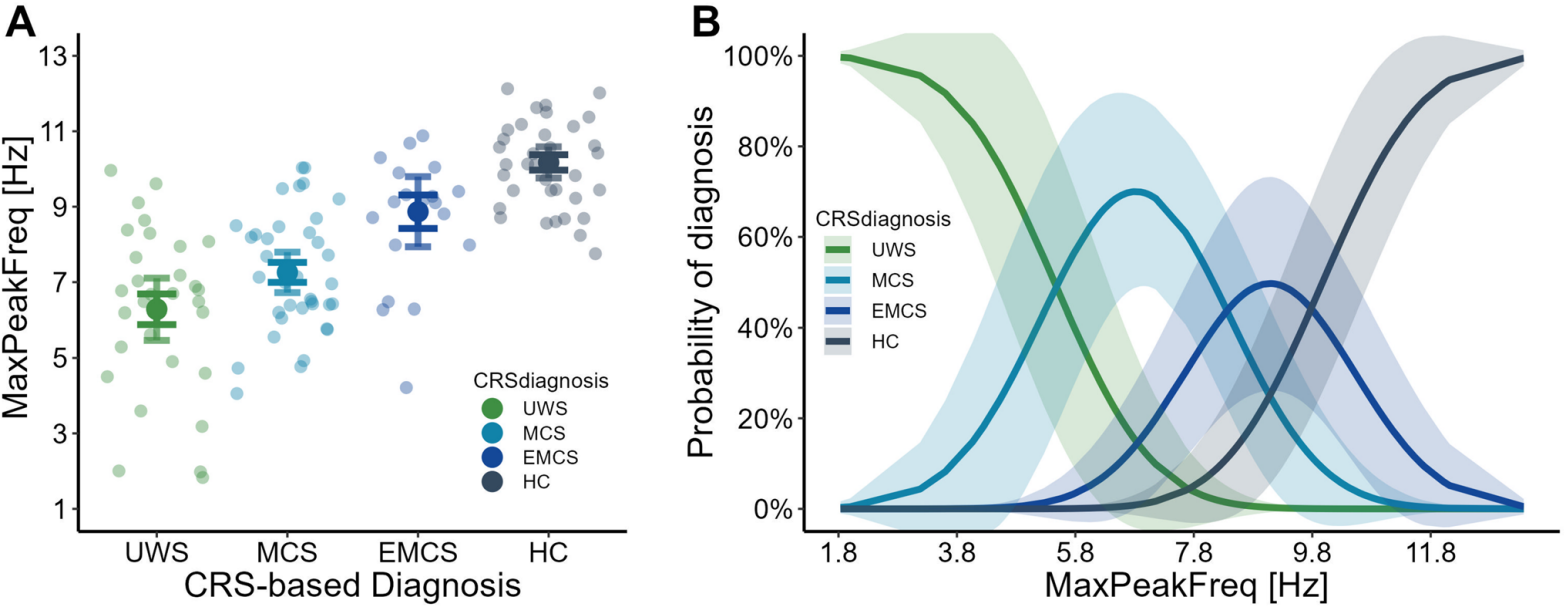
**A** The relationship between the frequency of the maximum peak in range 1–14 Hz (*MaxPeakFreq*) and CRS-based diagnosis including *UWS* (unresponsive wakefulness syndrome), *MCS* (minimally conscious state), *EMCS* (emergence from minimally conscious state) and *HC* (healthy control). *Light coloured dots* represent raw data points, *dark coloured dots* represent mean values for each diagnosis. Whiskers represent 95% SE (*dark coloured*) and 95% CE (*light coloured*) intervals. **B** Probability of classification to diagnosis groups (UWS, MCS, EMCS, HC) based on *MaxPeakFreq* (solid lines) based on the Model 2a fit. *Coloured ribbon* represents 95% SE around the fit (Color figure online)

Among all fitted *CRSdiagnosis* models, *Model 2a* performed best compared to null model (ΔLL = − 23.10, see Table 3). Analysis showed a statistically significant effect of the highest peak in the 1–14 Hz range (*MaxPeakFreq: β* = 0.75 ± 0.14, *CI:* [0.48 1.03], Fig. 3A). The probability of UWS classification decreases with the increasing *MaxPeakFreq* (*IQR* = 2.88) and approaches zero, with *MaxPeakFreq* value > 8.5 Hz. Probability distribution of MCS assignment presents a more gaussian-like shape, with the maximal frequency of the highest peak around 7 Hz, (*IQR* = 2.08)*.* For the *MaxPeakFreq* (*IQR* = 1.95) EMCS diagnosis probability increases only to the maximal point of 9 Hz, and starts decreasing. Finally, as predicted, HC profile presents an increase of *MaxPeakFreq (IQR* = 1.62) with the logarithmic increase of classification probability (Fig. 3B). MaxPeakFreq increased by 1 Hz multiplies the odds of HC diagnosis by 2.14. In the case of the AP gradient (*Model 2b*) and its interaction with the *MaxPeakFreq* (*Model 2c*) results did not reach significance threshold (*CI:* [− 1.25 4.57] and *CI:* [− 1.00 1.17], respectively, Table 3).

**Table 3:** Summary of the *CRSdiagnosis* models fitted with its accuracy diagnostic

Model	Effects	Estimate ± SE	z	p	95% CI	$\sigma^{2}$	$\tau^{00}$	LL
Model 2n	UWS | MCS	− 2.92 ± 0.74	− 3.95	** < 0.001**	**[**− **4.37 **− **1.47]**	1.00	12.14	− 148.59
	MCS | EMCS	− 0.39 ± 0.50	− 0.79	0.432	[− 1.36 0.58]			
	EMCS | HC	1.13 ± 0.49	2.28	**0.022**	**[0.16 2.10]**			
Model 2a	UWS | MCS	4.10 ± 0.93	4.39	** < 0.001**	**[2.27 5.94]**	1.00	2.84	− 125.49
	MCS | EMCS	6.18 ± 1.16	5.33	** < 0.001**	**[3.90 8.45]**			
	EMCS | HC	7.52 ± 1.38	5.44	** < 0.001**	**[4.81 10.22]**			
	MaxPeakFreq	0.75 ± 0.14	5.41	** < 0.001**	**[0.48 1.03]**			
Model 2b	UWS | MCS	− 2.76 ± 0.71	− 3.85	** < 0.001**	**[**− **4.17 **− **1.36]**	1.00	10.86	− 147.99
	MCS | EMCS	− 0.31 ± 0.47	− 0.66	0.510	[− 1.24 0.62]			
	EMCS | HC	1.15 ± 0.47	2.43	**0.015**	**[0.22 2.08]**			
	Gradient	1.66 ± 1.48	1.12	0.264	[− 1.25 4.57]			
Model 2c	UWS | MCS	4.23 ± 0.98	4.30	** < 0.001**	**[2.30 6.16]**	1.00	2.11	− 122.02
	MCS | EMCS	6.18 ± 1.19	5.19	** < 0.001**	**[3.85 8.52]**			
	EMCS | HC	7.47 ± 1.40	5.34	** < 0.001**	**[4.73 10.21]**			
	MaxPeakFreq: Gradient	0.09 ± 0.55	0.16	0.876	[− 1.00 1.17]			

Results that are significant (p < 0.05) are indicated in bold font

*UWS* unresponsive wakefulness syndrome, *MCS* minimally conscious state, *EMCS* emergence from MCS, *HC* healthy control, *LL* Log-Likelihood, $\sigma^\textit{2}$ variance of level-1 residual errors, $\tau^\textit{00}$ variance of level-2 residual errors

### The Influence of the Number of Detected Maxima on the Postulated Markers of Neurocognitive Recovery

**Fig. 4:**
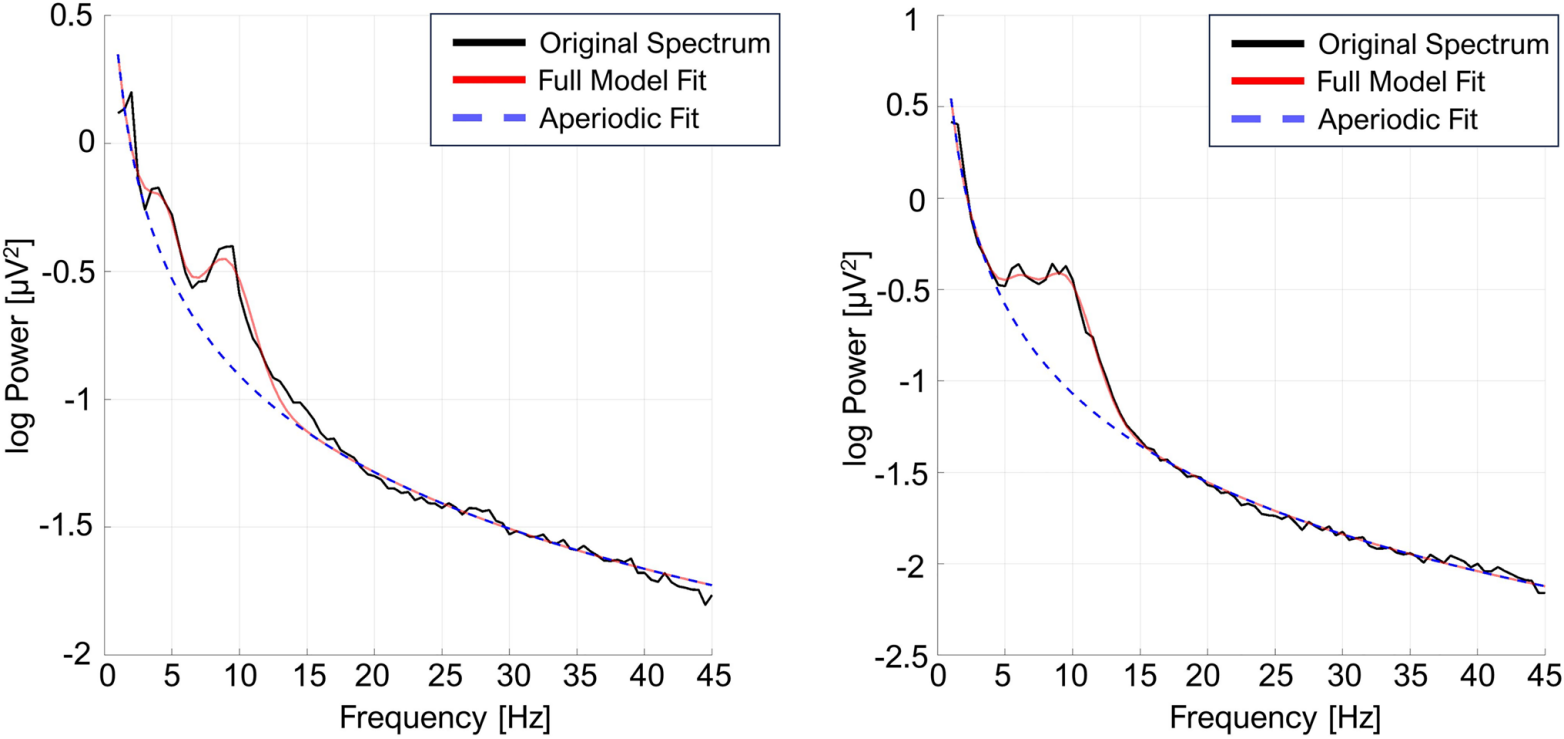
The examples of averaged power spectra of the *centro-parietal ROI* modeled with FOOOF parameters. *Black solid line* presents the original spectrum, whereas the *red solid* and *blue dashed lines* show full model fit and its aperiodic part, respectively. *Coloured ribbon* represents one standard error of the mean around the averaged fit. Presented spectra belong to the multiple peak (*left panel*) or single peak measurements (*right panel*) (Color figure online)

The power spectrum analysis of the resting-state data revealed diversity in the number of peaks detected in the 1–14 Hz range. To account for the possible relation between the number of detected peaks and the predictive power of our factors, we divided the dataset into two groups. The single peak group included measurements where the second maximal peak in the spectrum was not detectable, while the multiple peaks group included spectra with at least two detectable peaks in the 1–14 Hz range (demographic statistics in Table B1, Supplementary Material). The examples of both peak patterns are presented in Fig. 4. Analysis of the parameters of the second detected peak are not presented in the manuscript and the potential detection of the second peak was used only for data division. Both newly created groups were then subjected to the analysis, where MaxPeakFreq still represents the center frequency of the first detected peak with the highest amplitude within the 1–14 Hz search range and Gradient represents an AP gradient of the amplitude of this highest peak. All previously used models for *CRSscore* (*Model 1n/a/b/c*) and *CRSdiagnosis* (*Model 2n/a/b/c*) were refitted to both groups, separately (indicated as *‘s’* prefix in the model’s name for the single peak data and as ‘*m’* prefix for the multiple peak data).

The analysis revealed interesting differences between datasets. In the *CRSscore* models using a single-peak as well as multi-peak data, only the *MaxPeakFreq* factor reached a significance level (*p* = 0.003, see *Model s1a* in Table B2 of the Supplementary Materials and *p* = 0.010, see *Model m1a* in Table B3 of the Supplementary Material, respectively).

**Fig. 5:**
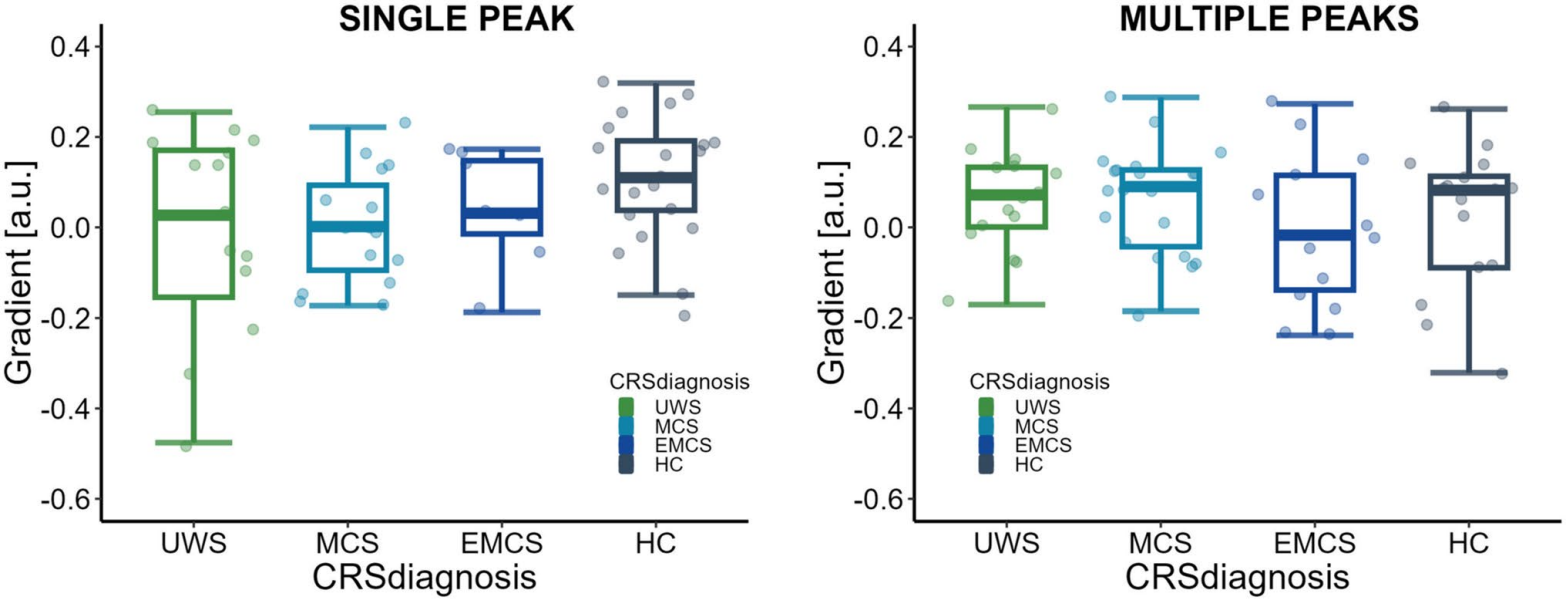
Changes in relationship between *Gradient* and *CRSdiagnosis* in a group of patients (*UWS* unresponsive wakefulness syndrome, *MCS* minimally conscious state, *EMCS* emergence from minimally conscious state, *HC* healthy control). Values of *Gradient* for different CRSdiagnosis with a single (*left panel*) and multiple (*right panel*) maximal peak in 1–14 Hz range. *Coloured horizontal bars* represent the mean, *whiskers* represent ± 1.5 × IQR (Color figure online)

However, in the *CRSdiagnosis* single-peak model, both *MaxPeakFreq* and *Gradient* presented a diagnostic potential, with *p* = 0.017 (see *Model s2a* in Table B2 of the Supplementary Material) and *p* = 0.018 (see Fig. 5 and *Model s2b* in Table A2 of the Supplementary Material), respectively. For the multi-peak data, again only the *MaxPeakFreq* variable maintained a high significance level (*p* < 0.001, see *Model m2a* in Table B3 of the Supplementary Materials). Dividing the dataset using the criterion of the number of maximal peaks in the searched EEG frequency range revealed a new aspect of the *Gradient* parameter. As demonstrated by the results for the patient spectra containing a single peak in the 1–14 Hz range, the antero-posterior gradient becomes a significant predictor of neurocognitive state (*Model s2b*). In such a case it seems that both the *Gradient* parameter and *MaxPeakFreq* are indicators of the same neurophysiological process.

To account for a possible influence of etiology and drug intake on our results we have conducted additional analyses with inclusion of etiology (anoxia, stroke, trauma). This analysis did not reveal any significant influence of that factor (see Appendix C of the Supplementary Materials for details). Furthermore, for a proportion of patients we were able to identify medications with possible influence on the central nervous system: Amantix (active substance *Amantadine Sulfate*), Baclofen (*Baclofen*) and Depakine (*Valproate*). An extended model which included those substances and patients without medications did not improve prediction capacity of any of the dependent variables (see Appendix D of the Supplementary Materials for details).

## Discussion

In this study, we have demonstrated that the frequency of the maximum peak in the EEG spectrum in the centro-parietal ROI is a good indicator of neurocognitive capacity in PDOC patients. We have found that the increase of the dominant EEG oscillation frequency within the 1–14 Hz frequency band predicts the increase of the total CRS-R score and the heightened probability of acquiring a more favorable diagnosis. Consequently, the slowing of the oscillations in the 1–14 Hz range is related to the less favorable PDOC diagnosis and the impairment of neurocognitive capacity indexed by the total CRS-R score.

For decomposition of the EEG spectral profiles, we used the FOOOF algorithm, which represents EEG spectra as a combination of two types of parameters. The first parameter, the aperiodic component, corresponds to the non-oscillatory aspect of the EEG signal appearing as the 1/f function over signal frequency. The second parameter, the oscillatory part of the EEG signal is fitted by FOOOF using the series of Gaussians with center frequencies representing the maximal amplitude of oscillation at a given frequency band. This way of decomposing the EEG signal in the frequency domain allows for a more flexible representation of the variability of the EEG spectrum in human populations. The FOOOF method is beneficial especially in comparison with the more dominant approach based on the analysis of mean power values calculated across traditional EEG spectral bands (eg. Lutkenhoff et al. 2020) which is based on a priori delimitation of the borders between EEG frequency bands.

While we have demonstrated that the primary oscillatory activity within the 1–14 Hz band can predict the neurocognitive state of the PDOC patient, we have also found that this primary activity can be confounded by additional neural activity in the same range that was manifested as additional peaks in the frequency spectrum. This activity may stem from the structural or functional cerebral lesions, which often manifest as slow or broadband oscillations within the theta or delta range (Galovic et al. 2017). Nevertheless, in the case of the EEG spectral profile marked by a single maximal peak in the 1–14 Hz range, we observed a relationship between the strength of AP gradient and CRS-R diagnosis. This trend was absent when considering the whole set of data, as well as when only the data with multiple maximal peaks was analyzed. Thus the success of the automatically assessed AP gradient value as a diagnostic factor depends on the integrity of the leading frequency in the EEG spectrum. Our results suggested that the existence of more than one peak in range 1–14 Hz weakens the contribution of the AP gradient. However, the relationship between *Gradient* and the frequency of the *MaxPeakFreq* suggests that both these indicators point at the single coherent neurophysiological process, which could be interpreted in terms of thalamo-cortical network connectivity. Previous research implies (Forgacs et al. 2014, 2017) that the presence of this kind of oscillation in the alpha range reflects preserved cell survival, so the observed slowing of the posterior oscillations is probably dependent on the inefficient facilitation of the thalamocortical connections by the neuromodulatory systems innervating the thalamus and the cortex.

On the basis of the observed relationships between spectral patterns and behavioral data (see Fig. 3), three types of spectral profiles can be identified, which correspond to a distinct modes of cerebral functioning in PDOC patients:

Type I: *the dominance of aperiodic EEG activity*. For this type, most of the signal energy is accumulated within delta range with a dominant profile 1/f in the spectrum. This spectral profile was observed mostly in the UWS group and can be interpreted as evidence for the low excitability of the cerebral cortex (Rosanova et al. 2012) and could be linked to the extensive functional and structural thalamocortical dysfunction. The dominance of delta-range activity has been previously reported in the studies of resting-state EEG in severe cases of brain lesions with UWS/VS diagnosis (eg. Lehembre et al. 2012; Lutkenhoff et al. 2020). However most of those studies were based on analyzing separate EEG spectral bands, so the 1/f pattern could be mistakenly treated as the increased oscillatory delta activity. The origins of aperiodic signals (1/f pattern) in human EEG are not well understood, however they are interpreted as the result of a shift in the balance between the synaptic excitation and inhibition (Donoghue et al. 2020; Gao et al 2017).

Type II: *the single theta-alpha oscillation* (4–14 Hz). This type is represented by a single oscillatory peak in the theta-alpha range within the centro-parietal ROI. Its frequency (the *MaxPeakFreq* parameter) is correlated with the PDOC diagnosis and total CRS-R score. Moreover, the gradient of the amplitude of this oscillation contributes to the prediction accuracy of the *MaxPeakFreq*. This pattern represents the strongest connection to the patient’s neurocognitive state as the frequency of oscillation, along with the prominence of AP gradient, most strongly predicts the PDOC diagnosis and CRS-R score in the group of patients where a single peak was detected within the 1–14 Hz range. Interestingly, it remained within 1–5 Hz range in UWS/VS patients, it ranged within the 5–8 Hz in MCS patients, and reached up to 8.5–10 Hz range in EMCS patients. In our opinion, despite its variable frequency, this oscillation represents a single neurophysiological process, related to the oscillation which, in normal awake EEG, is known as the posterior-dominant rhythm (PDR; St. Louis et al. 2016). PDR slowing can be observed under several conditions, but its mechanisms are not fully understood (O’Gorman et al. 2013). First, it is a normal developmental phenomenon that occurs during childhood (Marcuse et al. 2016; Rodriquez-Martinez et al. 2017; Cellier et al. 2021). Second, it has been shown to accompany various conditions of neurological pathology, such as mild traumatic injury (Galovic et al. 2017), white matter damage caused by vascular dementia (Moretti 2004) or decreased cerebral metabolism (Ingvar et al. 1976; O’Gorman et al. 2013). Finally it could be related to global brain pathology such as mild cognitive impairment or Alzheimer’s disease (Garces et al. 2013, Babiloni et al. 2015, Benz et al. 2014, Zimmermann et al. 2015, Dickinson et al. 2018).

Type III: *multiple delta-theta-alpha oscillations* (1–14 Hz). The characteristic feature of this type is a presence of multiple peaks within the delta-theta-alpha frequency range. This spectral pattern revealed weaker association with the patient’s neurocognitive state than the Type II. We consider this profile as a mixture of the Type II oscillation and other neural activity from the same range. If present, the other peaks are probably caused by the structural damage to the cortex or the underlying subcortical structures. The presence of local slow rhythms in the vicinity of the lesion has been observed repeatedly in previous studies (e.g. Sarasso et al. 2020). However, in the present study we do not have precise information about lesion location, so we cannot directly confirm this assumption. Note that the multiple peaks pattern was associated with weaker relation of *MaxPeakFreq* parameter and the clinical status (CRS-R) than single oscillation pattern. This suggests that the additional peaks are not related to the neural mechanisms predicting the clinical status of the PDOC patients, but are probably evoked by other types of brain pathology. Moreover, in the case of Type III the AP gradient data did not contribute to prediction of the state, confirming the presumption that they represent neural mechanisms which are of a different kind that those represented by the Type II. Further studies incorporating detailed information about the localization and etiology of brain injuries will advance our understanding of the confounding oscillatory activity not associated with the PDOC diagnosis. Consequently, this will help to improve our interpretation of the spectral profiles of PDOC patients.

An attempt to investigate possible features of resting-state EEG activity on the basis of EEG spectral profiles has been previously made by Forgacs et al. (Forgacs et al. 2014, 2017; Schiff et al. 2014) in anoxic PDOC patients. These authors distinguished four categories of frequency spectrum profiles in PDOC patients (*the ‘ABCD’ model*) and linked those to the anoxic patients' recovery. Crucially, in Forgacs et al. studies the spectral profiles were identified on the basis of qualitative experts evaluation. Their A-profile is very similar to our Type I profile, with a dominance of 1/f power distribution across frequencies and association with UWS/VS diagnosis. The B and D profiles identified by Forgacs et al. (2014) are characterized by a single theta band peak in the B-profile and a single alpha band peak for the D-profile (with an additional peak in the beta band). Forgacs et al. (2017) describe the B-profile in terms of spontaneous cortical oscillations with an absent thalamocortical input, while the D-profile is associated with restored thalamocortical connectivity and cortical oscillations driven by the tonic mode of thalamic firing. These two profiles overlap with our Type II profile, which is defined by the presence of a single peak spanning the combined theta-alpha range. In their studies these profiles most frequently appeared in MCS patients. The C-profile represented prominent peaks in theta in the posterior and beta activity in the frontal regions. Beta oscillations present in our sample were contaminated by frequent muscle artifacts in frontal channels. As we were not able to achieve the sufficient signal quality in the beta band, we could not corroborate Forgacs et al. (2014, 2017) findings within this frequency range.

In contrast with the distinctions proposed by Forgacs et al. (2014, 2017) we did not dissociate between spectral profiles with the single dominant theta and alpha activity, since our results suggest that they may represent a uniform neural mechanism in PDOC patients, based on the functional recovery of neuronal populations within the thalamocortical system. In this way, the process of regaining consciousness is based on the changes in dynamics of neural activity, resulting in the modulation of the frequency of the posterior dominant rhythm. The possible mechanism of this variability might involve dynamical changes in inhibition-excitation ratio within the thalamocortical system (Bhattacharya et al. 2011). We support our conjecture with the observation that the dominant rhythm in the theta-alpha range (the Type II spectral profile) was accompanied with the presence of the anterior–posterior gradient despite the frequency range that was lower than the “canonical” alpha range (namely 8–13 Hz).

Due to the numerous comorbidities, including epilepsy, parkinsonian tremor and muscle spasms, patients with disorders of consciousness are often treated with extensive pharmacological therapy. Some of those medications are reported to influence the EEG signal (Scarpino et al. 2019; Mecarelli et al. 2019; Ciurleo et al. 2013; Cho et al. 2012; Horiguchi et al. 1990; Marciani et al. 1993; Neckelmann et al. 1996; Salinsky et al. 2002). A particular pharmacotherapy is often linked to a specific etiology. Although etiology is not a direct indicator of a resulting disorder and a corresponding structural brain damage (Braakman et al. 1988; Levin et al. 1991; Levy et al. 1981; Sazbon et al. 1993; Sazbon and Groswasser 1990; Bates 1991; Shewmon and De Giorgio 1989), it was shown to have some impact on the neurocognitive state (Forgacs et al. 2020; Mecarelli et al. 2019; Forgacs et al. 2020; Schiff 2010). To account for the possibility of relation between measured EEG characteristics and patient’s etiology or received medication, we compared the final models with its versions taking the effect of patients *Etiology* (*anoxia*, *stroke* and *trauma*) and chosen *Medications* (*Amantix*, *Baclofen*, *Depakine*, *none*) into account. None of the analyses revealed a robust significance of the *Etiology* or *Medications* groups in *CRSscore* and *CRSdiagnosis* models (results presented in the Supplementary Material). This analysis ruled out etiology and drug intake as the significant interfering factors.

Finally, it should be also noted that in this experiment the CRS-R assessment was conducted only once. Currently multiple administration of CRS-R (at least five times during several days) is recommended by European and American Academies of Neurology (Giacino et al. 2018; Kondziella et al. 2020) in order to reduce the level of ambiguity in assessing the clinical condition of patients (Wannez et al. 2017). Thus, it can not be ruled out that its single usage might have weakened the estimates of the existing relationship between the clinical state of the patient and the measured spectral parameters. Moreover, we were not able to administer the CRS-R test on exactly the same day as the EEG measurement every time (the difference between CRS-R and EEG measurements in days is 1.91 ± 3.96). Though the result of CRS-R assessment tends to change over longer time periods, it may be expected to remain stable over several days (Beukema et al. 2016; Schiff et al. 2007).

Investigating individual power spectra based on extraction of frequency parameters within the 1–14 Hz range offers the possibility of complementing the diagnosis of the PDOC patient with a relatively simple tool. At the same time, however, the topographical patterns of dominant EEG oscillations should also be examined, as they could provide means of disentanglement between the EEG oscillations that have predictive power for patient state and other EEG oscillations that are also related to focal or global brain dysfunction, but did not show a clear relation to the diagnosis of PDOC.

## Conclusions

In this paper we investigated the possibility of improving the PDOC diagnostic process by introducing electrophysiological resting-state markers of patients' neurocognitive capacity, based on the posterior dominant peak frequency and evaluation of the anterior–posterior gradient of posterior oscillations. Both parameters stand as a promising diagnostic tool based on the qualitative approach used in other resting state studies in the PDOC population. We have identified three types of spectral profiles, linked with different brain pathology. Profiles were closely associated with distinct PDOC diagnoses. FOOOF algorithm of spectral decomposition, which controls for the aperiodic signal background, allowed us to efficiently capture characteristics of frequency distribution and observed differences between spectra with single and multiple maximal peaks.

Peak frequency and gradient were calculated automatically from the EEG signal. Our work complements previously established PDOC profiles by including various etiology types as well as healthy control samples for the comparison. It presents a viable proposal of a simple yet efficient method of distinguishing between spectral PDOC profiles and may provide the means to explore its underlying mechanisms.

### Supplementary Information

Below is the link to the electronic supplementary material.

Supplementary file1 (PDF 996 KB)

## Data Availability

The datasets generated during and/or analyzed during the current study are available from the corresponding author on reasonable request.

## References

[CR80] Agresti A (2002) Categorical Data Analysis. 2nd Edition, John Wiley & Sons, Inc., New York. 10.1002/0471249688

[CR1] Babiloni C, Percio CD, Boccardi M (2015). Occipital sources of resting-state alpha rhythms are related to local gray matter density in subjects with amnesic mild cognitive impairment and Alzheimer’s disease. Neurobiol Aging.

[CR3] Bai Y, Xia X, Li X (2017). A review of resting-state electroencephalography analysis in disorders of consciousness. Front Neurol.

[CR4] Bates D (1991). Defining prognosis in medical coma. J Neurol Neurosurg Psychiatry.

[CR100] Bates D, Mächler M, Bolker B, Walker S (2015) Fitting Linear Mixed-Effects Models Using lme4. J Stat Softw 67(1). 10.18637/jss.v067.i01

[CR6] Benz N, Hatz F, Bousleiman H (2014). Slowing of eeg background activity in Parkinson’s and Alzheimer’s disease with early cognitive dysfunction. Front Aging Neurosc.

[CR7] Beukema S, Gonzalez-Lara L, Finoia P (2016). A hierarchy of event-related potential markers of auditory processing in disorders of consciousness. Neuroimage Clin.

[CR8] Bhattacharya B, Coyle D, Maguire L (2011). A thalamo–cortico–thalamic neural mass model to study alpha rhythms in Alzheimer’s disease. Neural Netw.

[CR9] Binder M, Górska U, Wójcik-Krzemień A (2018). A validation of the polish version of the coma recovery scale-revised (crs-r). Brain Inj.

[CR10] Bodart O, Amico E, Gómez F (2018). Global structural integrity and effective connectivity in patients with disorders of consciousness. Brain Stimul.

[CR11] Braakman R, Jennett W, Minderhoud J (1988). Prognosis of the posttraumatic vegetative state. Acta Neurochir.

[CR13] Bürkner P, Vuorre M (2019). Ordinal regression models in psychology: a tutorial. Adv Methods Pract Psychol Sci.

[CR14] Cao B, Chen Y, Yu R (2019). Abnormal dynamic properties of functional connectivity in disorders of consciousness. NeuroImage: Clin.

[CR15] Cellier D, Riddle J, Petersen I (2021). The development of theta and alpha neural oscillations from ages 3 to 24 years. Develop Cognitive Neurosci.

[CR16] Childs N, Mercer W, Childs H (1993). Accuracy of diagnosis of persistent vegetative state. Neurology.

[CR17] Cho J, Koo D, Joo E (2012). Effect of levetiracetam monotherapy on background eeg activity and cognition in drug-naïve epilepsy patients. Clin Neurophysiol.

[CR19] Ciurleo R, Bramanti P, Calabrò R (2013). Pharmacotherapy for disorders of consciousness: are “awakening” drugs really a possibility?. Drugs.

[CR20] Demertzi A, Laureys S, Boly M, Banks W (2009). Coma, persistent vegetative states, diminished consciousness. Encyclopedia of Consciousness.

[CR21] Dickinson A, DiStefano C, Senturk D (2018). Peak alpha frequency is a neural marker of cognitive function across the autism spectrum. Eur J Neurosci.

[CR22] Donoghue T, Haller M, Peterson E (2020). Parameterizing neural power spectra into periodic and aperiodic components. Nat Neurosci.

[CR23] Estraneo A, Loreto V, Guarino I (2016). Standard eeg in diagnostic process of prolonged disorders of consciousness. Clin Neurophysiol.

[CR24] Forgacs P, Conte M, Fridman E (2014). Preservation of eeg organization in patients with impaired consciousness and imaging-based evidence of command-following. Ann Neurol.

[CR25] Forgacs P, Frey H, Velazquez A (2017). Dynamic regimes of neocortical activity linked to corticothalamic integrity correlate with outcomes in acute anoxic brain injury after cardiac arrest. Ann Clin Transl Neurol.

[CR26] Forgacs P, Devinsky O, Schiff N (2020). Independent functional outcomes after prolonged coma following cardiac arrest: a mechanistic hypothesis. Ann Neurol.

[CR27] Galovic M, Schmitz B, Tettenborn B (2017). C15eeg in inflammatory disorders, cerebrovascular diseases, trauma, and migraine. Niedermeyer’s electroencephalography: basic principles, clinical applications, and related fields.

[CR28] Gammel TC, Alkadaa LN, Saadon JR (2023). Brain circuitry of consciousness: a review of current models and a novel synergistic model with clinical application. Neurosurg Pract.

[CR29] Gao R, Peterson E, Voytek B (2017). Inferring synaptic excitation/inhibition balance from field potentials. Neuroimage.

[CR30] Garcés P, Vicente R, Wibral M (2013). Brain-wide slowing of spontaneous alpha rhythms in mild cognitive impairment. Front Aging Neurosci.

[CR31] Gennaro LD, Ferrara M, Curcio G (2001). Antero-posterior eeg changes during the wakefulness-sleep transition. Clin Neurophysiol.

[CR32] Giacino J, Ashwal S, Childs N (2002). The minimally conscious state: definition and diagnostic criteria. Neurology.

[CR33] Giacino J, Kalmar K, Whyte J (2004). The jfk coma recovery scale-revised: measurement characteristics and diagnostic utility. Arch Phys Med Rehabil.

[CR34] Giacino J, Schnakers C, Rodriguez-Moreno D (2009). Behavioral assessment in patients with disorders of consciousness: gold standard or fool’s gold?. Prog Brain Res.

[CR35] Giacino JT, Katz DI, Schiff ND (2018). Practice guideline update recommendations summary: disorders of consciousness. Neurology.

[CR36] Gosseries O, Vanhaudenhuyse A, Bruno M, Cvetkovic D, Cosic I (2011). Disorders of consciousness: coma, vegetative and minimally conscious states. States of consciousness.

[CR37] Gosseries O, Bruno M, Vanhaudenhuyse A, Tononi G, Boly M, Gosseries O (2016). Consciousness in the locked-in syndrome. The Neurology of Consciousness.

[CR38] Hirsch LJ, LaRoche SM, Gaspard N (2013). American clinical neurophysiology society’s standardized critical care EEG terminology: 2012 version. J Clin Neurophysiol.

[CR39] Hirsch LJ, Fong MWK, Leitinger M (2021). American clinical neurophysiology society’s standardized critical care EEG terminology: 2021 Version. J Clin Neurophysiol.

[CR40] Horiguchi J, Inami Y, Shoda T (1990). Effects of long-term amantadine treatment on clinical symptoms and eeg of a patient in a vegetative state. Clin Neuropharmacol.

[CR41] Ingvar D, Sjölund B, Ardö A (1976). Correlation between dominant eeg frequency, cerebral oxygen uptake and blood flow. Electroencephalogr Clin Neurophysiol.

[CR42] Kondziella D, Bender A, Diserens K (2020). European academy of neurology guideline on the diagnosis of coma and other disorders of consciousness. Eur J Neurol.

[CR43] Kremneva E, Legostaeva L, Morozova S (2019). Feasibility of non-Gaussian diffusion metrics in chronic disorders of consciousness. Brain Sci.

[CR44] Laureys S (2005). The neural correlate of (un)awareness: lessons from the vegetative state. Trends Cogn Sci.

[CR45] Laureys S, Celesia G, Cohadon F (2010). Unresponsive wakefulness syndrome: a new name for the vegetative state or apallic syndrome. BMC Med.

[CR46] Lechinger J, Bothe K, Pichler G (2013). Crs-r score in disorders of consciousness is strongly related to spectral eeg at rest. J Neurol.

[CR47] Lehembre R, Bruno M, Vanhaudenhuyse A (2012). Resting-state eeg study of comatose patients: a connectivity and frequency analysis to find differences between vegetative and minimally conscious states. Funct Neurol.

[CR48] Levin H, Saydjari C, Eisenberg H (1991). Vegetative state after closed-head injury. a traumatic coma data bank report. Archives of Neurology.

[CR49] Levy D, Bates D, Caronna J (1981). Prognosis in nontraumatic coma. Ann Intern Med.

[CR50] Louis ES, Frey L (2016) Electroencephalography (EEG): an introductory text and atlas of normal and abnormal findings in adults, children, and infants. American Epilepsy Society, Chicago, IL,. 10.5698/978-0-9979756-0-4 27748095

[CR51] Lutkenhoff E, Nigri A, Sebastiano D (2020). Eeg power spectra and subcortical pathology in chronic disorders of consciousness. Psychol Med.

[CR52] Marciani M, Gigli G, Stefanini F (1993). Effect of carbamazepine on eeg background activity and on interictal epileptiform abnormalities in focal epilepsy. Int J Neurosci.

[CR53] Marcuse L, Fields M, Yoo J (2016). Rowan’s Primer of EEG.

[CR54] Mecarelli O, Brienza M, Grippo A (2019). Disorders of consciousness. Clinical electroencephalography.

[CR55] Monti M, Laureys S, Owen A (2010). The vegetative state. BMJ.

[CR56] Moretti D (2004). Individual analysis of eeg frequency and band power in mild Alzheimer’s disease. Clin Neurophysiol.

[CR57] Moscatelli A, Mezzetti M, Lacquaniti F (2012). Modeling psychophysical data at the population-level: the generalized linear mixed model. J vis.

[CR58] Neckelmann D, Bjorvatn B, Bjørkum A (1996). Citalopram: differential sleep/wake and eeg power spectrum effects after single dose and chronic administration. Behav Brain Res.

[CR59] O’Gorman R, Poil S, Brandeis D (2013). Coupling between resting cerebral perfusion and eeg. Brain Topogr.

[CR60] Piarulli A, Bergamasco M, Thibaut A (2016). Eeg ultradian rhythmicity differences in disorders of consciousness during wakefulness. J Neurol.

[CR61] Pistoia F, Sacco S, Franceschini M (2014). Comorbidities: a key issue in patients with disorders of consciousness. J Neurotrauma.

[CR62] Posner J, Saper C, Schiff N (2019). Plum and Posner’s Diagnosis of Stupor and Coma.

[CR63] Rodríguez-Martínez E, Ruiz-Martínez F, Paulino CB (2017). Frequency shift in topography of spontaneous brain rhythms from childhood to adulthood. Cogn Neurodyn.

[CR64] Rosanova M, Gosseries O, Casarotto S (2012). Recovery of cortical effective connectivity and recovery of consciousness in vegetative patients. Brain.

[CR65] Salinsky M, Binder M, Oken B (2002). Effects of gabapentin and carbamazepine on the eeg and cognition in healthy volunteers. Epilepsia.

[CR66] Sarasso S, D’Ambrosio S, Fecchio M (2020). Local sleep-like cortical reactivity in the awake brain after focal injury. Brain.

[CR67] Sazbon L, Groswasser Z (1990). Outcome in 134 patients with prolonged post-traumatic unawareness. J Neurosurg.

[CR68] Sazbon L, Zagreba F, Ronen J (1993). Course and outcome of patients in vegetative state of nontraumatic etiology. J Neurol Neurosurg Psychiatry.

[CR69] Scarpino M, Lolli F, Hakiki B (2019). Prognostic value of post-acute eeg in severe disorders of consciousness using american clinical neurophysiology society terminology. Neurophysiol Clin.

[CR70] Schiff N (2010). Recovery of consciousness after brain injury: a mesocircuit hypothesis. Trends Neurosci.

[CR71] Schiff N, Giacino J, Kalmar K (2007). Behavioral improvements with thalamic stimulation after severe traumatic brain injury. Nature.

[CR72] Schiff N, Nauvel T, Victor J (2014). Large-scale brain dynamics in disorders of consciousness. Curr Opin Neurobiol.

[CR73] Schnakers C (2020). Update on diagnosis in disorders of consciousness. Expert Rev Neurother.

[CR74] Schnakers C, Vanhaudenhuyse A, Giacino J (2009). Diagnostic accuracy of the vegetative and minimally conscious state: clinical consensus versus standardized neurobehavioral assessment. BMC Neurol.

[CR75] Schnakers C, Edlow B, Chatelle C, Laureys S, Gosseries O, Tononi G (2016). Chapter 11 - minimally conscious state. The neurology of consciousness.

[CR76] Seel RT, Sherer M, Whyte J, Katz DI, Giacino JT, Rosenbaum AM, Hammond FM, Kalmar K, Pape TL, Zafonte R, Biester RC, Kaelin D, Kean J, Zasler N, American Congress of Rehabilitation Medicine, Brain Injury-Interdisciplinary Special Interest Group, Disorders of Consciousness Task Force (2010). Assessment scales for disorders of consciousness: evidence-based recommendations for clinical practice and research. Arch Phys Med Rehabil..

[CR77] Shewmon D, Giorgio CD (1989). Early prognosis in anoxic coma. Reliability and rationale. Neurol Clin.

[CR78] Snijders T, Bosker R (2012). Multilevel analysis: an introduction to basic and advanced multilevel modeling.

[CR79] Stender J, Gosseries O, Bruno M (2014). Diagnostic precision of pet imaging and functional mri in disorders of consciousness: a clinical validation study. Lancet.

[CR81] Wannez S, Heine L, Thonnard M (2017). The repetition of behavioral assessments in diagnosis of disorders of consciousness. Ann Neurol.

[CR82] Van Erp WS, Lavrijsen JCM, van de Laar FA, Vos PE, Laureys S, Koopmans RTCM (2014). The vegetative state/unresponsive wakefulness syndrome: a systematic review of prevalence studies. Eur J Neurol.

[CR85] Zimmermann R, Gschwandtner U, Hatz F (2015). Correlation of eeg slowing with cognitive domains in nondemented patients with Parkinson’s disease. Dement Geriatr Cogn Dis.

