# Supplementary material for: Distinct Spectral Profiles of Awake Resting EEG in Disorders of Consciousness: The Role of Frequency and Topography of Oscillations
Source: Brain Topogr. 2023 Dec 29;37(1):138–51. doi: 10.1007/s10548-023-01024-0 (PMC10771586; doi:10.1007/s10548-023-01024-0)
Supplement: Supplementary file 1 — Supplementary file1 (PDF 996 KB) [file 10548_2023_1024_MOESM1_ESM.pdf]

Supplementary Material

Appendix A

Clinical and demographic characteristics of patients.

Table A1 Clinical and demographic characteristics of patients.

Patient	Sex	Etiology	Session	Age	Time since injury [months]	CRS-R measurement date	EEG measurement date	AS	VS	MS	OS	CS	ARS	CRS score	Diagnosis	Baclofen	Amantix	Depakine	Exclusion criteria
P01	M	anoxia	1	18	38	2015-05-22	2015-05-25	2	0	2	0	0	2	6	UWS	N/A	N/A	N/A	artifacts in the midline data
P02	M	anoxia	1	66	18	2015-05-11	2015-05-27	2	4	5	2	1	2	16	MCS	N/A	N/A	N/A	
			2	66	23	2015-10-09	2015-09-29	3	5	2	2	1	2	15	MCS	0	0	0	
			3	67	30	2016-05-05	2016-05-10	3	5	2	2	1	2	15	MCS	N/A	N/A	N/A	artifacts in the midline data
			4	67	31	2016-06-06	2016-06-07	3	5	2	2	1	2	15	MCS	N/A	N/A	N/A	extensive artifacts in the midline data
			5	68	39	2017-01-31	2017-01-31	2	3	5	2	0	3	15	MCS	0	0	0	extensive artifacts in the midline data
P03	F	N/A	1	43	8	2015-05-20	2015-05-26	2	3	2	2	1	2	12	MCS	N/A	N/A	N/A	
			2	44	16	2016-01-21	2016-01-27	2	3	2	1	0	1	9	MCS	1	0	0	
P04	M	trauma	1	65	12	2015-05-20	2015-05-26	1	3	2	2	2	2	12	MCS	N/A	N/A	N/A	
			2	65	16	2015-10-06	2015-09-29	1	2	1	1	0	1	6	UWS	N/A	N/A	N/A	artifacts in the midline data
P05	M	trauma	1	58	8	2015-05-20	2015-05-27	2	0	0	0	0	2	4	UWS	N/A	N/A	N/A	
P06	F	trauma	1	51	18	2015-06-08	2015-05-28	4	5	5	2	2	3	21	EMCS	N/A	N/A	N/A	
P07	M	anoxia	1	26	17	2015-10-08	2015-09-30	1	0	1	0	0	2	4	UWS	N/A	N/A	N/A	
P08	M	trauma	1	31	6	2015-06-08	2015-05-29	3	3	5	2	2	3	18	MCS	1	0	0	
			2	31	10	2015-10-07	2015-09-30	3	5	2	1	1	3	15	MCS	1	0	0	
P09	F	trauma	1	21	16	2015-05-22	2015-05-29	4	5	5	2	1	1	18	MCS	N/A	N/A	N/A	artifacts in the midline data
P10	M	anoxia	1	37	22	2015-07-03	2015-06-24	1	0	1	0	0	2	4	UWS	N/A	N/A	N/A	artifacts in the midline data
			2	38	29	2016-02-05	2016-02-05	2	1	2	0	0	2	7	UWS	1	0	1	
P11	M	anoxia	1	48	108	2016-01-19	2016-01-25	1	1	0	0	0	1	3	UWS	N/A	N/A	N/A	artifacts in the midline data
P12	M	anoxia	1	51	67	2015-07-03	2015-06-25	1	0	1	1	0	1	4	UWS	N/A	N/A	N/A	artifacts in the midline data
			2	52	76	2016-01-20	2016-01-26	1	0	1	0	0	1	3	UWS	1	0	0	
P13	M	anoxia	1	39	50	2015-07-03	2015-06-26	1	0	2	1	0	1	5	UWS	N/A	N/A	N/A	
			2	40	57	2016-01-22	2016-01-28	1	0	2	0	0	1	4	UWS	0	0	0	
P14	M	anoxia	1	59	8	2016-01-19	2016-01-25	1	0	1	0	0	1	3	UWS	1	0	0	
P15	M	anoxia	1	27	67	2015-07-01	2015-06-26	3	3	2	2	1	3	14	MCS	N/A	N/A	N/A	
			2	28	80	2016-01-04	2016-01-27	4	5	5	1	2	3	20	EMCS	1	0	0	
			3	28	86	2016-06-06	2016-06-06	4	5	6	2	2	3	22	EMCS	N/A	N/A	N/A	
P16	M	anoxia	1	51	17	2015-10-09	2015-10-01	1	0	0	0	0	2	3	UWS	N/A	N/A	N/A	artifacts in the midline data
			2	52	24	2016-05-10	2016-05-09	1	0	1	0	0	1	3	UWS	N/A	N/A	N/A	artifacts in the midline data
			3	52	25	2016-06-07	2016-06-07	1	0	1	0	0	1	3	UWS	N/A	N/A	N/A	artifacts in the midline data
			4	53	32	2017-01-31	2017-01-31	1	0	1	0	0	1	3	UWS	0	0	0	
P17	F	trauma	1	24	15	2015-10-09	2015-10-02	1	0	2	1	0	1	5	UWS	0	0	1	extensive artifacts in the midline data
			2	25	22	2016-05-09	2016-05-10	1	0	1	1	0	1	4	UWS	N/A	N/A	N/A	
			3	25	23	2016-06-08	2016-06-08	1	0	1	1	0	1	4	UWS	N/A	N/A	N/A	
P18	M	N/A	1	28	6	2016-02-05	2016-01-29	1	0	1	0	0	1	3	UWS	1	0	1	artifacts in the midline data
			2	28	11	2016-06-07	2016-06-07	1	0	1	0	0	0	2	UWS	N/A	N/A	N/A	artifacts in the midline data
P19	F	stroke	1	37	5	2016-02-05	2016-01-29	4	5	4	1	2	2	18	EMCS	0	1	0	artifacts in the midline data
			2	37	13	2016-10-03	2016-10-03	4	5	2	1	2	3	17	EMCS	N/A	N/A	N/A	
			3	38	18	2017-02-01	2017-02-01	4	5	2	1	2	3	17	EMCS	0	0	1	
P20	M	trauma	1	37	11	2016-05-06	2016-05-11	1	0	1	0	0	0	2	UWS	N/A	N/A	N/A	
			2	37	16	2016-10-05	2016-10-05	1	0	1	0	0	1	3	UWS	N/A	N/A	N/A	
P21	M	trauma	1	21	8	2016-05-16	2016-05-11	4	5	2	2	1	1	15	MCS	N/A	N/A	N/A	
P22	M	trauma	1	19	6	2016-05-09	2016-05-12	4	5	1	2	2	2	16	MCS	N/A	N/A	N/A	
P23	M	N/A	1	29	6	2016-05-17	2016-05-12	1	0	2	0	0	2	5	UWS	N/A	N/A	N/A	
			2	29	11	2016-10-05	2016-10-05	1	0	3	1	0	2	7	MCS	N/A	N/A	N/A	
			3	30	15	2017-01-30	2017-01-30	1	3	3	1	0	2	10	MCS	1	0	1	
			4	30	23	2017-10-05	2017-10-05	1	1	1	1	0	1	5	UWS	N/A	N/A	N/A	
P24	F	trauma	1	25	6	2016-05-13	2016-05-13	1	1	1	0	0	2	5	UWS	N/A	N/A	N/A	
			2	26	19	2017-10-02	2017-10-02	1	1	2	1	0	2	7	UWS	N/A	N/A	N/A	
			3	26	23	2019-08-09	2019-08-09	1	0	1	0	0	1	3	UWS	N/A	N/A	N/A	
			4	28	45	2019-10-25	2019-10-25	3	1	2	1	0	2	9	MCS	0	0	0	
			5	28	46	2019-12-23	2019-12-23	1	3	2	1	1	2	10	MCS	0	0	0	
			6	28	48	2019-10-25	2019-10-25	1	1	2	1	0	2	7	UWS	N/A	N/A	N/A	artifacts in the midline data
			7	28	50	2019-12-23	2019-12-23	3	1	2	1	0	2	9	MCS	N/A	N/A	N/A	
P25	M	anoxia	1	37	10	2016-05-12	2016-05-13	2	0	1	0	0	2	5	UWS	N/A	N/A	N/A	extensive artifacts in the midline data
			2	37	15	2016-10-03	2016-10-03	1	0	1	0	0	2	4	UWS	N/A	N/A	N/A	artifacts in the midline data
P26	F	stroke	1	38	8	2016-10-04	2016-10-04	4	5	1	1	2	3	16	EMCS	N/A	N/A	N/A	
P27	M	trauma	1	55	14	2016-06-07	2016-06-08	4	5	4	1	2	3	19	EMCS	N/A	N/A	N/A	
			2	56	22	2017-01-31	2017-01-30	4	5	4	1	1	3	18	MCS	0	0	0	
P28	F	trauma	1	30	8	2016-06-07	2016-06-08	1	1	1	1	0	2	6	UWS	N/A	N/A	N/A	
P29	M	anoxia	1	33	19	2016-10-04	2016-10-04	1	0	1	0	0	1	3	UWS	N/A	N/A	N/A	artifacts in the midline data
			2	34	23	2017-02-02	2017-02-02	1	0	1	0	0	1	3	UWS	1	1	0	extensive artifacts in the midline data
			3	34	23	2017-02-02	2017-02-02	1	0	1	0	0	1	3	UWS	1	0	1	extensive artifacts in the midline data
P30	M	trauma	1	65	3	2016-10-17	2016-10-17	3	4	2	1	1	1	12	MCS	1	1	0	
P31	M	trauma	1	65	32	2016-10-17	2016-10-17	0	1	2	2	0	2	7	UWS	N/A	N/A	N/A	artifacts in the midline data
			2	66	40	2017-05-22	2017-05-22	1	0	2	2	0	2	7	UWS	N/A	N/A	N/A	
P32	M	anoxia	1	47	6	2016-10-24	2016-10-24	3	0	1	1	1	1	7	MCS	0	1	0	artifacts in the midline data
P33	F	anoxia	1	23	2	2016-10-24	2016-10-24	3	1	2	1	1	3	11	MCS	N/A	N/A	N/A	extensive artifacts in the midline data
P34	F	anoxia	1	18	2	2016-11-26	2016-11-26	1	0	2	1	0	1	5	UWS	1	0	0	artifacts in the midline data
P35	M	anoxia	1	80	4	2016-11-26	2016-11-26	1	0	1	1	0	1	4	UWS	0	0	0	artifacts in the midline data
			2	81	9	2017-05-22	2017-05-22	1	0	1	1	0	1	4	UWS	N/A	N/A	N/A	extensive artifacts in the midline data
P36	F	trauma	1	52	2	2016-12-05	2016-12-05	3	1	5	1	0	2	12	MCS	0	0	0	
P37	M	trauma	1	18	36	2017-01-16	2017-01-16	1	1	2	1	0	1	6	UWS	0	0	1	
P38	M	anoxia	1	37	8	2017-01-23	2017-01-23	1	0	2	1	0	2	6	UWS	N/A	N/A	N/A	extensive artifacts in the midline data
P39	M	anoxia	1	11	21	2017-01-23	2017-01-23	2	2	5	2	0	2	13	MCS	N/A	N/A	N/A	extensive artifacts in the midline data
P40	F	anoxia	1	62	2	2017-05-30	2017-05-30	1	0	2	1	0	1	5	UWS	N/A	N/A	N/A	artifacts in the midline data
			2	62	3	2017-10-04	2017-10-04	1	0	2	1	0	1	5	UWS	N/A	N/A	N/A	extensive artifacts in the midline data
P41	F	trauma	1	43	1	2017-02-03	2017-02-03	1	1	1	0	0	1	4	UWS	N/A	N/A	N/A	artifacts in the midline data
			2	43	2	2017-06-01	2017-06-01	1	1	2	1	0	1	6	UWS	N/A	N/A	N/A	
P42	F	stroke	1	49	1	2017-02-21	2017-02-21	1	3	2	1	1	1	9	MCS	N/A	N/A	N/A	
P43	M	trauma	1	21	1	2017-02-21	2017-02-21	3	5	2	1	2	3	16	EMCS	N/A	N/A	N/A	
			2	21	2	2017-05-29	2017-05-29	3	3	5	1	2	3	17	EMCS	N/A	N/A	N/A	

			3	21	3	2017-11-08	2017-11-08	4	5	6	1	1	3	20	EMCS	0	0	0	
P44	F	trauma	1	31	1	2017-02-22	2017-02-22	3	5	2	1	1	2	14	MCS	N/A	N/A	N/A	
			2	31	2	2017-05-30	2017-05-30	2	3	1	1	0	2	9	MCS	N/A	N/A	N/A	
P45	F	stroke	1	75	1	2017-02-22	2017-02-22	2	3	5	3	1	1	15	MCS	N/A	N/A	N/A	
P46	M	trauma	1	20	1	2017-03-27	2017-03-27	0	0	2	1	0	1	4	UWS	N/A	N/A	N/A	artifacts in the midline data
P47	F	trauma	1	23	1	2017-03-27	2017-03-27	1	2	2	1	0	1	7	MCS	N/A	N/A	N/A	
P48	F	stroke	1	31	1	2017-04-10	2017-04-10	3	1	5	2	1	3	15	MCS	N/A	N/A	N/A	
			2	32	2	2018-03-16	2018-03-15	3	4	6	2	1	2	18	EMCS	0	0	0	
			3	32	3	2018-03-16	2018-03-16	3	4	6	2	1	2	18	EMCS	N/A	N/A	N/A	
P49	M	anoxia	1	34	1	2017-04-10	2017-04-10	1	1	1	1	0	2	6	UWS	N/A	N/A	N/A	artifacts in the midline data
P50	M	trauma	1	25	1	2017-05-30	2017-05-29	2	3	2	1	0	2	10	MCS	N/A	N/A	N/A	
			2	25	2	2017-10-03	2017-10-03	2	3	2	2	0	2	11	MCS	1	0	0	
			3	25	3	2017-11-07	2017-11-07	2	3	2	1	0	2	10	MCS	1	0	0	
P51	M	trauma	1	22	1	2017-05-29	2017-05-29	1	1	1	1	0	1	5	UWS	N/A	N/A	N/A	
			2	23	2	2018-03-12	2018-03-12	1	1	2	1	0	1	6	UWS	1	0	1	
P52	M	trauma	1	24	1	2017-05-30	2017-05-30	4	5	6	3	1	3	22	EMCS	N/A	N/A	N/A	
P53	F	trauma	1	37	1	2017-05-31	2017-05-31	3	3	1	1	0	3	11	MCS	1	1	0	extensive artifacts in the midline data
			2	37	2	2017-11-08	2017-11-08	4	5	2	1	2	3	17	EMCS	1	1	0	
			3	38	3	2018-03-12	2018-03-12	3	3	2	2	0	2	12	MCS	1	1	0	
P54	M	trauma	1	34	1	2017-06-01	2017-06-01	1	0	1	1	0	2	5	UWS	N/A	N/A	N/A	artifacts in the midline data
P55	F	trauma	1	35	1	2017-06-01	2017-06-01	1	0	1	1	0	2	5	UWS	N/A	N/A	N/A	artifacts in the midline data
P56	F	N/A	1	23	1	2017-06-19	2017-06-19	1	1	1	2	0	2	7	UWS	N/A	N/A	N/A	artifacts in the midline data
P57	M	trauma	1	30	1	2017-06-19	2017-06-19	1	1	2	1	0	2	7	UWS	N/A	N/A	N/A	
P58	F	trauma	1	32	1	2017-10-02	2017-10-02	4	5	2	2	1	3	17	MCS	1	1	0	
			2	32	2	2017-11-08	2017-11-08	4	5	6	3	2	3	23	EMCS	1	1	1	
P59	M	trauma	1	30	1	2017-10-02	2017-10-02	3	3	2	1	0	1	10	MCS	0	1	0	
			2	30	2	2017-11-07	2017-11-07	3	5	2	1	1	1	13	MCS	0	1	0	
P60	M	trauma	1	26	1	2017-10-03	2017-10-03	4	5	5	1	2	3	20	EMCS	0	0	0	
P61	F	trauma	1	22	1	2017-10-04	2017-10-04	2	3	2	1	0	2	10	MCS	N/A	N/A	N/A	extensive artifacts in the midline data
			2	22	2	2017-11-07	2017-11-07	2	3	2	1	0	2	10	MCS	N/A	N/A	N/A	extensive artifacts in the midline data
P62	M	anoxia	1	28	1	2017-10-05	2017-10-05	1	1	2	1	0	2	7	UWS	N/A	N/A	N/A	artifacts in the midline data
P63	M	anoxia	1	33	1	2017-10-05	2017-10-05	1	0	1	0	0	2	4	UWS	N/A	N/A	N/A	artifacts in the midline data
P64	M	trauma	1	55	1	2018-02-09	2018-02-09	0	3	2	0	0	1	6	MCS	1	0	0	
P65	M	trauma	1	30	1	2018-03-12	2018-03-12	1	0	2	2	0	1	6	UWS	0	0	0	
P66	F	stroke	1	63	1	2018-03-13	2018-03-12	2	1	2	2	0	1	8	UWS	N/A	N/A	N/A	extensive artifacts in the midline data
P67	F	anoxia	1	37	1	2018-03-13	2018-03-12	1	4	6	2	0	3	16	EMCS	1	0	1	extensive artifacts in the midline data
P68	M	trauma	1	38	1	2018-03-16	2018-03-16	1	0	2	1	0	1	5	UWS	1	0	1	
P69	F	stroke	1	32	1	2018-04-17	2018-04-17	2	1	2	1	0	1	7	UWS	0	0	1	
P70	M	anoxia	1	59	1	2018-04-18	2018-04-27	1	0	0	2	0	2	5	UWS	0	0	0	artifacts in the midline data
P71	F	anoxia	1	56	1	2018-09-11	2018-09-11	1	0	1	0	0	1	3	UWS	N/A	N/A	N/A	extensive artifacts in the midline data
P72	F	stroke	1	N/A	1	2018-11-28	2018-11-28	3	0	5	1	1	1	11	MCS	0	0	0	
P73	M	trauma	1	41	1	2018-11-30	2018-11-30	3	0	2	1	0	1	7	MCS	1	0	0	
P74	M	stroke	1	55	1	2018-11-30	2018-11-30	1	0	2	0	0	2	5	UWS	1	0	1	
P75	M	anoxia	1	39	2	2019-08-09	2019-08-09	1	0	2	0	0	1	4	UWS	0	0	0	
			2	39	3	2019-09-17	2019-09-17	1	0	2	1	0	1	5	UWS	0	0	0	
P76	F	anoxia	1	41	1	2019-09-04	2019-09-04	1	0	1	1	1	2	6	UWS	N/A	N/A	N/A	artifacts in the midline data
P77	M	trauma	1	N/A	1	2019-11-04	2019-11-04	4	5	5	0	3	3	20	EMCS	0	0	0	
			2	N/A	2	2019-12-03	2019-12-03	4	5	6	0	2	3	20	EMCS	0	0	0	
P78	F	stroke	1	53	1	2019-11-04	2019-11-04	1	2	2	0	1	3	7	MCS	0	0	0	
			2	53	2	2019-12-03	2019-12-03	1	1	3	0	1	3	7	MCS	0	0	0	
P79	F	trauma	1	N/A	1	2019-11-04	2019-11-04	4	5	6	0	2	3	20	EMCS	0	0	0	
			2	N/A	2	2019-12-03	2019-12-03	4	5	6	0	3	2	20	EMCS	0	0	0	

UWS unresponsive wakefulness syndrome, *MCS* minimally conscious state, *EMCS* emergence from minimally conscious state, *F* female, *M* male, *AS* auditory subscale, *VS* verbal subscale, *MS* motor subscale, *OS* oromotor/verbal subscale, *CS* communication subscale, *ARS* arousal subscale.

Appendix B

The influence of the number of detected maxima on the postulated markers of neurocognitive recovery.

Table B1 Demographic features of patient groups within numbers of maximal peaks in the 1-14 Hz range.

Number of maximal peaks in 1-14 Hz range						
Groups		*N measurements*	*N*	*Females*	*Age*	
					M	*SD*
Model 1	single peak	37	30	12	37.12	13.11
	multiple peaks	49	33	11	33.62	13.28
Model 2	single peak	58	51	24	32.62	12.62
	multiple peaks	65	49	21	36.71	13.94

Table B2 Results describing *CRSscore* and *CRSdiagnosis* models in a group of patients with single maximal peak in 1-14 Hz range.

Model	*Effects*	*Estimate ± SE*	*t*	*p*	*95% CI*	σ^2	τ_{00}	*LL*
Model s1n	Intercept	10.47 ± 1.06	9.84	**<0.001**	**[8.39 12.38]**	5.59	29.01	-113.70
Model s1a	Intercept	1.76 ± 2.88	0.61	0.546	[-3.76 7.76]	3.47	25.23	-109.29
	MaxPeakFreq	1.19 ± 0.37	3.21	**0.003**	**[0.42 1.92]**			
Model s1b	Intercept	10.51 ± 1.06	9.93	**<0.001**	**[8.46 12.54]**	5.93	28.11	-113.64
	Gradient	1.39 ± 3.63	0.38	0.705	[-6.46 8.62]			
Model s1c	Intercept	0.23 ± 2.90	0.08	0.938	[-5.10 6.17]	4.26	19.07	-106.80
	MaxPeakFreq : Gradient	1.76 ± 1.08	1.63	0.114	[-0.34 4.07]			
Model	*Effects*	*Estimate ± SE*	*z*	*p*	*95% CI*	σ^2	τ_{00}	*LL*
Model s2n	UWS | MCS	-2.18 ± 0.91	-2.39	**0.017**	**[-3.97 -0.39]**	1.00	9.54	-73.30
	MCS | EMCS	-0.13 ± 0.57	-0.22	0.825	[-1.24 0.99]			
	EMCS | HC	0.86 ± 0.61	1.41	0.157	[-0.33 2.056]			
Model s2a	UWS | MCS	15.85 ± 6.92	2.29	**0.022**	**[2.29 29.43]**	1.00	15.58	-52.43
	MCS | EMCS	19.93 ± 8.24	2.42	**0.016**	**[3.78 36.08]**			
	EMCS | HC	22.17 ± 9.16	2.42	**0.016**	**[4.21 40.13]**			
	MaxPeakFreq	2.37 ± 0.99	2.38	**0.017**	**[0.42 4.31]**			
Model s2b	UWS | MCS	-1.50 ± 0.75	-2.00	**0.046**	**[-2.96 -0.03]**	1.00	3.97	-71.59
	MCS | EMCS	0.05 ± 0.40	0.13	0.900	[-0.74 0.88]			
	EMCS | HC	0.78 ± 0.46	1.71	0.088	[-0.12 1.68]			
	Gradient	3.35 ± 1.65	2.03	**0.018**	**[0.11 6.59]**			
Model s2c	UWS | MCS	16.27 ± 6.87	2.37	**0.011**	**[2.80 29.74]**	1.00	14.44	-47.33
	MCS | EMCS	20.42 ± 7.98	2.56	**0.011**	**[4.77 36.07]**			
	EMCS | HC	22.86 ± 8.99	2.54	**0.013**	**[5.25 40.48]**			
	MaxPeakFreq : Gradient	2.87 ± 1.64	1.74	0.081	[-0.35 6.10]			

UWS unresponsive wakefulness syndrome, *MCS* minimally conscious state, *EMCS* emergence from MCS, *HC* healthy control, *LL* Log-Likelihood, σ^2 variance of level-1 residual errors, τ_{00} variance of level-2 residual errors. Results that are significant ($p < 0.05$) are indicated in bold font.

Table B3 Results describing *CRSscore* and *CRSdiagnosis* models in a group of patients with multiple maximal peaks in the 1-14 Hz range.

Model	*Effects*	*Estimate ± SE*	*t*	*p*	*95% CI*	σ^2	τ_{00}	*LL*
Model m1n	Intercept	11.21 ± 1.05	10.62	**<0.001**	**[9.04 13.55]**	5.81	32.21	-148.05
Model m1a	Intercept	4.58 ± 2.65	1.73	0.090	[-0.89 9.42]	6.59	23.62	-145.02
	MaxPeakFreq	0.89 ± 0.33	2.68	**0.010**	**[0.23 1.60]**			
Model m1b	Intercept	11.24 ± 1.06	10.55	**<0.001**	**[9.04 13.40]**	5.85	32.01	-148.03
	Gradient	-0.89 ± 5.16	-0.17	0.864	[-10.54 9.33]			
Model m1c	Intercept	-0.51 ± 3.51	-0.14	0.886	[-7.53 7.45]	5.87	22.09	-142.85
	MaxPeakFreq : Gradient	-4.16 ± 2.21	-1.88	0.067	[-8.49 0.52]			
Model	*Effects*	*Estimate ± SE*	*z*	*p*	*95% CI*	σ^2	τ_{00}	*LL*
Model m2n	UWS | MCS	-2.98 ± 1.03	-2.89	**0.004**	**[-5.00 -0.96]**	1.00	13.71	-80.51
	MCS | EMCS	-0.14 ± 0.67	-0.21	0.830	[-1.46 1.17]			
	EMCS | HC	1.90 ± 0.81	2.34	**0.019**	**[0.31 3.50]**			
Model m2a	UWS | MCS	3.65 ± 1.34	2.72	**0.007**	**[1.02 6.29]**	1.00	3.34	-70.58
	MCS | EMCS	5.86 ± 1.70	3.46	**0.001**	**[2.54 9.19]**			
	EMCS | HC	7.49 ± 2.06	3.63	**<0.001**	**[3.45 11.53]**			
	MaxPeakFreq	0.71 ± 0.20	3.51	**<0.001**	**[0.31 1.11]**			
Model m2b	UWS | MCS	-3.03 ± 1.05	-2.89	**0.004**	**[-5.08 -0.98]**	1.00	13.74	-80.41
	MCS | EMCS	-0.18 ± 0.68	-0.26	0.789	[-1.51 1.15]			
	EMCS | HC	1.88 ± 0.81	2.31	**0.021**	**[0.28 3.48]**			
	Gradient	-1.22 ± 2.87	-0.43	0.670	[-6.86 4.40]			
Model m2c	UWS | MCS	5.16 ± 1.89	2.73	**0.006**	**[1.46 8.86]**	1.00	4.21	-69.21
	MCS | EMCS	7.58 ± 2.32	3.27	**0.001**	**[3.04 12.12]**			
	EMCS | HC	9.36 ± 2.71	3.45	**0.001**	**[4.04 14.68]**			
	MaxPeakFreq : Gradient	-1.56 ± 1.21	-1.28	0.200	[-3.94 0.82]			

UWS unresponsive wakefulness syndrome, *MCS* minimally conscious state, *EMCS* emergence from MCS, *HC* healthy control, *LL* Log-Likelihood, σ^2 variance of level-1 residual errors, τ_{00} variance of level-2 residual errors. Results that are significant ($p < 0.05$) are indicated in bold font.

Figure B1 Changes in relationship between *MaxPeakFreq*, *Gradient*, *CRSscore* and *CRSdiagnosis* in a group of patients (UWS - unresponsive wakefulness syndrome, MCS - minimally conscious state, EMCS - emergence from minimally conscious state, HC - healthy control) with single (*left panels*) or multiple (*right panels*) maximal peaks in 1-14 Hz range. **A** Relationship between *MaxPeakFreq* and *Gradient*. *Dark coloured dots* represent mean value for UWS, MCS and EMCS groups. *Whiskers* represent 95% SE intervals. **B** Values of *MaxPeakFreq* for different *CRSdiagnosis*. *Coloured horizontal bars* represent the mean, *whiskers* represent ± 1.5 * IQR.

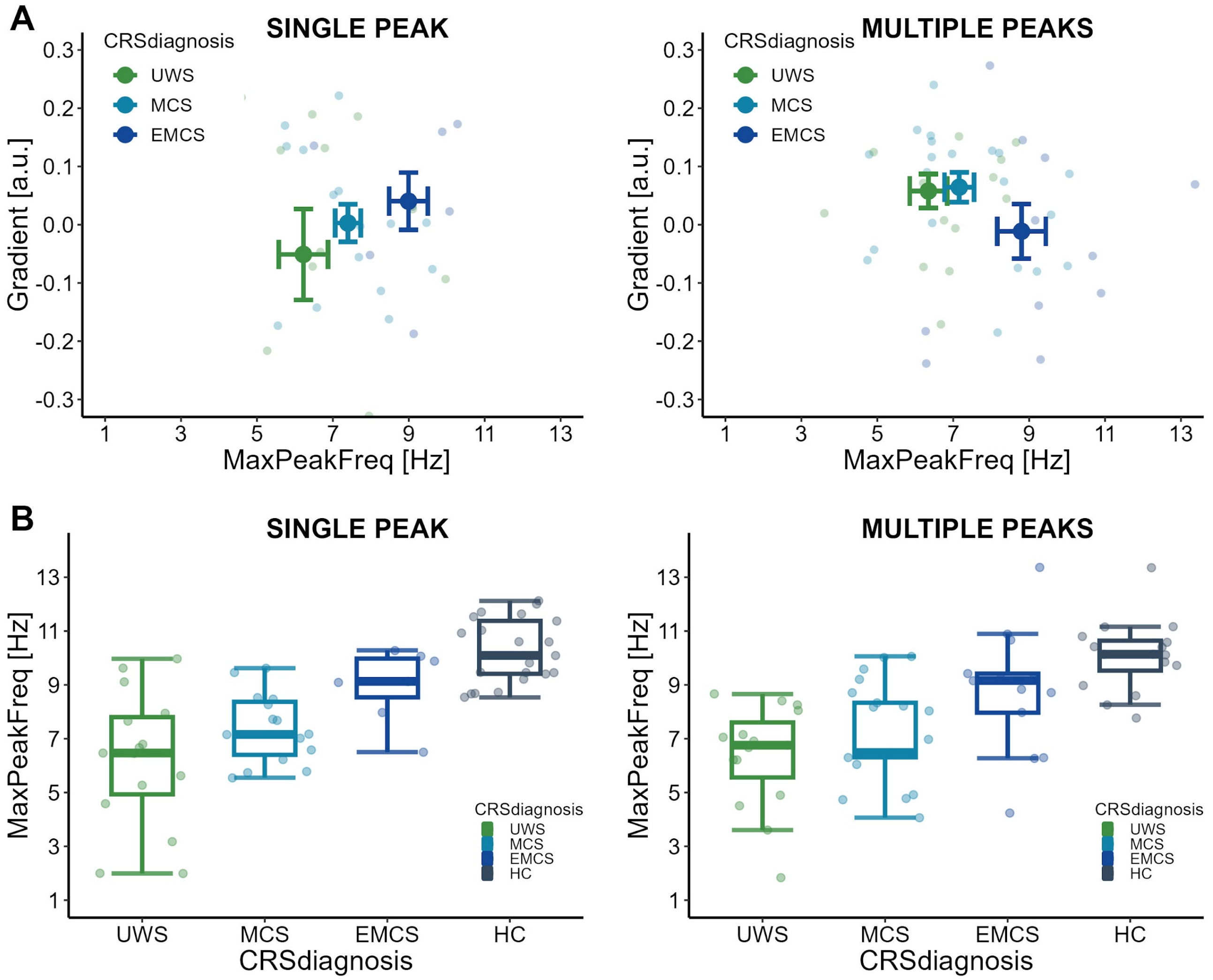

Appendix C

Controlling for the influence of etiology on the markers of PDOC patients state.

To account for the possibility of relation between measured EEG characteristics and patient's etiology (Forgacs, Devinsky & Schiff, 2020; Schiff, 2010), we selected a group of 51 patients (80 measurements), who were classified to one of the following etiologies: anoxia, stroke and trauma (demographic statistics in Table C1, Supplementary materials). None of the analyses revealed significant differences between *Etiology* groups for *CRSscore* and *CRSdiagnosis* variables (see *Model 1aE* and *Model 2aE* in Table C2, Supplementary materials). Furthermore, none of the etiologies improved the relation between both variables and the MaxpeakFreq (see *Model 1cE* and *Model 2cE* in Table C2, Supplementary materials) or Gradient (see *Model 1cE* and *Model 2cE* in Table C3, Supplementary materials). We therefore ruled out the possible relation of patients etiology and the improvement of a neurocognitive state in our research (see Forgacs, Devinsky, Schiff, 2020).

Table C1 Demographic features of groups of patients within chosen *Etiology*.

Etiology					
Groups	*N measurements*	*N*	*Females*	*Age*	
				M	*SD*
anoxia	14	9	0	42.86	14.06
stroke	13	9	8	43.75	13.45
trauma	53	33	11	33.82	13.10

Table C2 *CRSscore* and *CRSdiagnosis* models with *MaxPeakFreq* and *Etiology*.

Model	*Effects*	*Estimate ± SE*	*t*	*p*	*95% CI*	σ^2	τ_{00}	*LL*
Model 1nE	Intercept	10.72 ± 0.83	12.94	**<0.001**	**[9.09 12.29]**	4.85	31.34	-235.76
Model 1aE	trauma: stroke	-0.17 ± 2.21	-0.08	0.997	[-5.50 5.16]	4.81	28.82	-233.71
	trauma: anoxia	4.28 ± 2.21	1.94	0.138	[-1.04 9.60]			
	stroke: anoxia	4.45 ± 2.77	1.60	0.252	[-2.23 11.12]			
Model 1bE	Intercept	5.53 ± 1.82	3.03	**<0.003**	**[2.01 9.45]**	4.74	26.10	-231.22
	MaxPeakFreq	0.71 ± 0.23	3.14	**<0.003**	**[0.21 1.14]**			
Model 1cE	MaxPeakFreq : trauma	0.76 ± 0.43	1.78	0.078	[-0.09 1.62]	4.69	24.58	-229.71
	MaxPeakFreq : stroke	0.62 ± 0.47	1.32	0.194	[-0.33 1.57]			
	MaxPeakFreq : anoxia	0.60 ± 0.37	1.63	0.109	[-0.14 1.34]			
Model	*Effects*	*Estimate ± SE*	*z*	*p*	*95% CI*	σ^2	τ_{00}	*LL*
Model 2nE	UWS | MCS	-0.80 ± 0.54	-1.47	0.143	[-1.86 0.27]	1.00	8.13	-74.12
	MCS | EMCS	2.41 ± 0.88	2.73	**0.006**	**[0.68 4.15]**			
Model 2aE	UWS | MCS	3.50 ± 1.44	2.43	**0.015**	**[0.68 6.32]**	1.00	5.24	-67.59
	MCS | EMCS	6.54 ± 1.91	3.43	**0.008**	**[2.80 10.28]**			
	MaxPeakFreq	0.58 ± 0.20	2.96	**0.003**	**[0.20 0.97]**			
Model 2bE	UWS | MCS	-1.08 ± 0.59	-1.82	0.069	[-2.24 0.08]	1.00	6.19	-70.98
	MCS | EMCS	1.97 ± 0.74	2.65	**0.008**	**[0.51 3.42]**			
	trauma: stroke	-0.79 ± 1.10	-0.72	0.753	[-3.36 1.79]			
	trauma: anoxia	2.71 ± 1.36	1.99	0.114	[-0.48 5.91]			
	stroke: anoxia	3.50 ± 1.67	2.10	0.090	[-0.41 7.41]			
Model 2cE	UWS | MCS	1.47 ± 1.74	0.84	0.399	[-1.95 4.89]	1.00	5.51	-61.79
	MCS | EMCS	4.86 ± 1.95	2.50	**0.013**	**[1.04 8.68]**			
	MaxPeakFreq : trauma	0.37 ± 0.23	1.60	0.109	[-0.08 0.82]			
	MaxPeakFreq : stroke	2.42 ± 1.12	2.16	**0.030**	**[0.23 4.62]**			
	MaxPeakFreq : anoxia	0.73 ± 0.43	1.70	0.089	[-0.11 1.58]			

UWS unresponsive wakefulness syndrome, *MCS* minimally conscious state, *EMCS* emergence from MCS, *HC* healthy control, *LL* Log-Likelihood, σ^2 variance of level-1 residual errors, τ_{00} variance of level-2 residual errors. Results that are significant ($p < 0.05$) are indicated in bold font.

Table C3 *CRSscore* and *CRSdiagnosis* models with *Gradient* and *Etiology*.

Model	*Effects*	*Estimate ± SE*	*t*	*p*	*95% CI*	σ^2	τ_{00}	*LL*
Model 1nE	Intercept	10.72 ± 0.83	12.94	**<0.001**	**[9.07 12.28]**	4.85	31.34	-235.76
Model 1aE	trauma: stroke	-0.17 ± 2.21	-0.08	0.997	[-5.50 5.16]	4.81	28.82	-233.71
	trauma: anoxia	4.28 ± 2.21	1.94	0.138	[-1.04 9.60]			
	stroke: anoxia	4.45 ± 2.77	1.60	0.252	[-2.23 11.12]			
Model 1bE	Intercept	10.72 ± 0.83	12.92	**<0.001**	**[9.09 12.37]**	4.84	31.40	-235.76
	Gradient	-0.31 ± 2.61	-0.12	0.905	[-5.79 4.94]			
Model 1cE	Gradient : trauma	-2.52 ± 4.37	-0.58	0.567	[-11.29 6.25]	4.77	28.23	-233.09
	Gradient : stroke	-4.64 ± 6.34	-0.73	0.469	[-17.45 8.18]			
	Gradient : anoxia	2.13 ± 4.24	0.50	0.618	[-6.33 10.58]			
Model	*Effects*	*Estimate ± SE*	*z*	*p*	*95% CI*	σ^2	τ_{00}	*LL*
Model 2nE	UWS | MCS	-0.76 ± 0.49	-1.53	0.125	[-1.73 0.21]	1.00	6.86	-74.34
	MCS | EMCS	2.25 ± 0.75	2.99	**0.003**	**[0.77 3.72]**			
Model 2aE	UWS | MCS	-0.76 ± 0.50	-1.53	0.126	[-1.73 0.21]	1.00	6.95	-74.33
	MCS | EMCS	2.26 ± 0.76	2.97	**0.003**	**[0.77 3.75]**			
	Gradient	-0.22 ± 1.79	-0.12	0.901	[-3.72 3.28]			
Model 2bE	UWS | MCS	-1.08 ± 0.59	-1.82	0.069	[-2.24 0.08]	1.00	6.19	-70.98
	MCS | EMCS	1.97 ± 0.74	2.65	**0.008**	**[0.51 3.42]**			
	trauma: stroke	-0.79 ± 1.10	-0.72	0.753	[-3.36 1.79]			
	trauma: anoxia	2.71 ± 1.36	1.99	0.114	[-0.48 5.91]			
	stroke: anoxia	3.50 ± 1.67	2.10	0.090	[-0.41 7.41]			
Model 2cE	UWS | MCS	-1.17 ± 0.63	-1.86	0.062	[-2.40 0.06]	1.00	6.95	-68.66
	MCS | EMCS	2.08 ± 0.78	2.66	**0.008**	**[0.54 3.61]**			
	Gradient : trauma	-1.35 ± 2.86	-0.47	0.638	[-6.95 4.25]			
	Gradient : stroke	-8.73 ± 6.56	-1.33	0.183	[-21.58 4.12]			
	Gradient : anoxia	4.97 ± 4.51	1.10	0.270	[-3.87 13.82]			

UWS unresponsive wakefulness syndrome, *MCS* minimally conscious state, *EMCS* emergence from MCS, *HC* healthy control, *LL* Log-Likelihood, σ^2 variance of level-1 residual errors, τ_{00} variance of level-2 residual errors. Results that are significant ($p < 0.05$) are indicated in bold font.

Appendix D

Controlling for the influence of drug intake on the markers of PDOC patients state.

To account for the possibility of relation between measured EEG characteristics and received medication (*Depakine*, Zenkov 2002; *Amantix*, Terzano et al. 1983; *Baclofen*, Badr et al. 1983; Ciurleo, Bramanti, Calabrò, 2013) we selected a group of 36 patients (46 measurements), for whose this information was available: Amantix, Baclofen, Depakine, and finally no medication of interest. (demographic statistics in Table D1, Supplementary materials).

None of the analyses revealed significant differences between *Medications* groups for *CRSscore* and *CRSdiagnosis* variables (see *Model 1aM* and *Model 2aM* in Table D2, Supplementary materials). Furthermore, none of the etiologies improved the relation between both variables and the MaxpeakFreq (see *Model 1cM* and *Model 2cM* in Table D2, Supplementary materials) or Gradient (see *Model 1cM* and *Model 2cM* in Table D3, Supplementary materials). Therefore, in our opinion, drug intake did not serve as an interfering factor.

Table D1 Demographic features of groups of patients within chosen *Medication*.

Medications					
Groups	*N measurements*	*N*	*Females*	*Age*	
				M	*SD*
no drugs	10	7	3	40.83	14.39
Amantix	7	4	2	37.71	12.45
Baclofen	20	16	3	38.95	12.29
Depakine	9	9	3	33.78	10.54

Table D2 *CRSscore* and *CRSdiagnosis* models with *MaxPeakFreq* and *Medications*.

Model	*Effects*	*Estimate ± SE*	*t*	*p*	*95% CI*	σ^2	τ_{00}	*LL*
Model 1nM	Intercept	9.92 ± 1.18	8.40	**<0.001**	**[7.43 12.57]**	4.83	24.58	-101.58
Model 1aM	Intercept	-2.37 ± 2.71	-0.87	0.388	[-7.40 2.95]	2.11	22.15	-93.29
	MaxPeakFreq	1.72 ± 0.35	4.93	**<0.001**	**[1.00 2.36]**			
Model 1bM	Amantix : Baclofen	0.23 ± 1.39	0.17	0.985	[-3.28 3.74]	4.78	24.64	-101.51
	Amantix : Depakine	-0.16 ± 1.72	-0.10	0.995	[-4.49 4.16]			
	Baclofen : Depakine	-0.40 ± 1.24	-0.32	0.995	[-3.51 2.71]			
Model 1cM	MaxPeakFreq : Amantix	2.32 ± 0.69	3.36	**0.003**	**[0.89 3.75]**	1.86	22.14	-92.22
	MaxPeakFreq : Baclofen	1.40 ± 0.49	2.87	**0.006**	**[0.41 2.39]**			
	MaxPeakFreq : Depakine	1.63 ± 0.60	2.73	**0.009**	**[0.42 2.83]**			
Model	*Effects*	*Estimate ± SE*	*z*	*p*	*95% CI*	σ^2	τ_{00}	*LL*
Model 2nM	UWS | MCS	-0.85 ± 0.93	-0.92	0.357	[-2.67 0.96]	1.00	10.44	-30.36
	MCS | EMCS	3.52 ± 1.69	2.08	**0.037**	**[0.21 6.83]**			
Model 2aM	Model did not converge.							
Model 2bM	UWS | MCS	-0.92 ± 1.15	-0.80	0.423	[-3.17 1.33]	1.00	10.39	-30.35
	MCS | EMCS	3.45 ± 1.99	1.73	0.084	[-0.46 7.35]			
	Amantix : Baclofen	0.09 ± 0.81	0.11	0.993	[-1.80 1.98]			
	Amantix : Depakine	0.04 ± 1.18	0.04	0.999	[-2.73 2.81]			
	Baclofen : Depakine	-0.05 ± 1.00	-0.05	0.999	[-2.39 2.29]			
Model 2cM	Model did not converge.							

UWS unresponsive wakefulness syndrome, *MCS* minimally conscious state, *EMCS* emergence from MCS, *HC* healthy control, *LL* Log-Likelihood, σ^2 variance of level-1 residual errors, τ_{00} variance of level-2 residual errors. Results that are significant ($p < 0.05$) are indicated in bold font.

Table D3 *CRSscore* and *CRSdiagnosis* models with *Gradient* and *Medications*.

Model	*Effects*	*Estimate ± SE*	*t*	*p*	*95% CI*	σ^2	τ_{00}	*LL*
Model 1nM	Intercept	9.92 ± 1.18	8.40	**<0.001**	**[7.47 12.03]**	4.83	24.58	-101.58
Model 1aM	Intercept	10.00 ± 1.16	8.59	**<0.001**	**[7.71 12.42]**	4.78	24.64	-101.32
	Gradient	3.15 ± 4.32	0.73	0.472	[-5.79 12.67]			
Model 1bM	Amantix : Baclofen	0.23 ± 1.39	0.17	0.985	[-3.28 3.74]	4.89	23.52	-101.51
	Amantix : Depakine	-0.16 ± 1.72	-0.10	0.995	[-4.49 4.16]			
	Baclofen : Depakine	-0.40 ± 1.24	-0.32	0.945	[-3.51 2.71]			
Model 1cM	Gradient : Amantix	3.56 ± 10.61	0.34	0.740	[-18.38 25.50]	4.88	23.35	-101.24
	Gradient : Baclofen	3.21 ± 5.17	0.62	0.538	[-7.27 13.70]			
	Gradient : Depakine	1.76 ± 7.50	0.23	0.815	[-13.37 16.90]			
Model	*Effects*	*Estimate ± SE*	*z*	*p*	*95% CI*	σ^2	τ_{00}	*LL*
Model 2nM	UWS | MCS	-0.85 ± 0.93	-0.92	0.357	[-2.67 0.96]	1.00	10.44	-30.36
	MCS | EMCS	3.52 ± 1.69	2.08	**0.037**	**[0.21 6.83]**			
Model 2aM	UWS | MCS	-0.86 ± 0.87	-0.99	0.323	[-2.56 0.84]	1.00	7.85	-29.99
	MCS | EMCS	3.15 ± 1.60	1.97	**0.049**	**[0.01 6.30]**			
	Gradient	2.62 ± 2.92	0.90	0.370	[-3.11 8.34]			
Model 2bM	UWS | MCS	-0.92 ± 1.15	-0.80	0.423	[-3.17 1.33]	1.00	10.39	-30.35
	MCS | EMCS	3.45 ± 1.99	1.73	0.084	[-0.46 7.35]			
	Amantix : Baclofen	0.09 ± 0.81	0.11	0.994	[-1.80 1.98]			
	Amantix : Depakine	0.04 ± 1.18	0.04	0.999	[-2.73 2.81]			
	Baclofen : Depakine	-0.05 ± 1.00	-0.05	0.999	[-2.39 2.29]			
Model 2cM	UWS | MCS	-1.03 ± 1.02	-1.01	0.311	[-3.04 0.97]	1.00	6.27	-29.66
	MCS | EMCS	2.71 ± 1.89	1.44	0.150	[-0.98 6.41]			
	Gradient : Amantix	0.20 ± 5.65	0.04	0.971	[-10.88 11.30]			
	Gradient : Baclofen	3.78 ± 3.38	1.12	0.262	[-2.83 10.40]			
	Gradient : Depakine	0.78 ± 5.12	0.15	0.879	[-9.27 10.80]			

UWS unresponsive wakefulness syndrome, *MCS* minimally conscious state, *EMCS* emergence from MCS, *HC* healthy control, *LL* Log-Likelihood, σ^2 variance of level-1 residual errors, τ_{00} variance of level-2 residual errors. Results that are significant ($p < 0.05$) are indicated in bold font.

References

Badr G, Matousek M, Frederiksen P (1983) A quantitative EEG analysis of the effects of baclofen on man. Neuropsychobiology 10(1):13–18. https://doi.org/10.1159/000117978

Ciurleo R, Bramanti P, Calabrò R (2013) Pharmacotherapy for disorders of consciousness: Are "awakening" drugs really a possibility? Drugs 73(17):1849–1862. https://doi.org/10.1007/s40265-013-0138-8

Forgacs P, Devinsky O, Schiff N (2020) Independent functional outcomes after prolonged coma following cardiac arrest: A mechanistic hypothesis. Annals of Neurology 87(4):618–632. https://doi.org/10.1002/ana.25690

Terzano M, Montanari E, Calzetti S, et al (1983) The effect of amantadine on arousal and EEG patterns in Creutzfeldt-Jakob Disease. Archives of neurology 40(9):555–559. https://doi.org/10.1001/archneur.1983.04050080055010

Schiff N (2010) Recovery of consciousness after brain injury: a mesocircuit hypothesis. Trends Neurosci 33(1):1–9. https://doi.org/10.1016/j.tins.2009.11.002

Zenkov L (2002) Clinical importance of eeg changes during the treatment of epilepsy with valproate (depakene chrono). Zhurnal nevrologii i psikhiatrii imeni SS Korsakova 102(3):20–26
